# Bacterial Indicator of Agricultural Management for Soil under No-Till Crop Production

**DOI:** 10.1371/journal.pone.0051075

**Published:** 2012-11-30

**Authors:** Eva L. M. Figuerola, Leandro D. Guerrero, Silvina M. Rosa, Leandro Simonetti, Matías E. Duval, Juan A. Galantini, José C. Bedano, Luis G. Wall, Leonardo Erijman

**Affiliations:** 1 Instituto de Investigaciones en Ingeniería Genética y Biología Molecular (INGEBI-CONICET) Vuelta de Obligado 2490, Buenos Aires, Argentina; 2 CERZOS-CONICET Departamento de Agronomía, Universidad Nacional del Sur, Bahía Blanca, Argentina; 3 Departamento de Geología, Universidad Nacional de Río Cuarto, Río Cuarto, Córdoba, Argentina; 4 Departamento de Ciencia y Tecnología, Universidad Nacional de Quilmes, Roque Sáenz Peña 352, Bernal, Argentina; 5 Facultad de Ciencias Exactas y Naturales, Universidad de Buenos Aires, Ciudad Universitaria, Pabellón 2, Buenos Aires, Argentina; Wageningen University, The Netherlands

## Abstract

The rise in the world demand for food poses a challenge to our ability to sustain soil fertility and sustainability. The increasing use of no-till agriculture, adopted in many areas of the world as an alternative to conventional farming, may contribute to reduce the erosion of soils and the increase in the soil carbon pool. However, the advantages of no-till agriculture are jeopardized when its use is linked to the expansion of crop monoculture. The aim of this study was to survey bacterial communities to find indicators of soil quality related to contrasting agriculture management in soils under no-till farming. Four sites in production agriculture, with different soil properties, situated across a west-east transect in the most productive region in the Argentinean pampas, were taken as the basis for replication. Working definitions of Good no-till Agricultural Practices (GAP) and Poor no-till Agricultural Practices (PAP) were adopted for two distinct scenarios in terms of crop rotation, fertilization, agrochemicals use and pest control. Non-cultivated soils nearby the agricultural sites were taken as additional control treatments. Tag-encoded pyrosequencing was used to deeply sample the 16S rRNA gene from bacteria residing in soils corresponding to the three treatments at the four locations. Although bacterial communities as a whole appeared to be structured chiefly by a marked biogeographic provincialism, the distribution of a few taxa was shaped as well by environmental conditions related to agricultural management practices. A statistically supported approach was used to define candidates for management-indicator organisms, subsequently validated using quantitative PCR. We suggest that the ratio between the normalized abundance of a selected group of bacteria within the GP1 group of the phylum Acidobacteria and the genus *Rubellimicrobium* of the Alphaproteobacteria may serve as a potential management-indicator to discriminate between sustainable *vs*. non-sustainable agricultural practices in the Pampa region.

## Introduction

Sowing crop into no-till soil is a farming method that has initially been developed as an alternative to conventional tillage practices, with the aims of using less fossil fuels, reducing the erosion of soils, and increasing the soil carbon pool [1]. Soil structure can be significantly modified through reduced-till management practices [2]. Soil aggregates are less subjected to dry and wet cycles in no-tilled soil, compared to conventional-tilled soil, due to the protection exerted by surface residues. Therefore, it appears that reduced-till management reduces the risk of surface runoff, increase soil aggregation, and improve soil hydrological properties [3]. This is particularly true if no-till management is combined with diverse crop rotation [4].

Additional driving forces for no-till agriculture are the lower production costs, the higher yields and the incorporation of less fertile areas into crop production [4]. During the past several decades, no-till agriculture has been increasingly adopted in many areas of the world [5]. In Argentina, this practice has spread steadily in the last 30 years [6], covering presently almost 20 million hectare, which represents 70% of the total cultivated area [4]. Through the adoption of this novel agriculture management, farmers have been gradually incorporated novel technologies for weed, disease and fertilizer management through trial-and-error learning. The combination of these technologies with no-till management led to a farmers’ definition of good agricultural practices on the basis of economic yield alongside soil conservation and gain in productivity. Yet this situation rapidly highlighted the need for new working hypotheses to aid in soil quality monitoring.

Driven by the influence of favorable market conditions, a substantial portion of that area is presently dedicated to soybean monoculture, often combined with minimal nutrient restoration. From the noticeable increase in soil born diseases caused by residue- and soil-inhabiting pathogens selected by the previous crop, questions arise about the ability to maintain soil fertility and sustainability if monoculture prevails over the crop rotation [7].

The use of soil quality indicators is important in order to guide land and resource management decisions. Traditionally, soil quality research has focused primarily on soil chemical and physical properties [8]. In general, assessment of soil quality will be influenced by management factors, and by climate and soil type as well. In view of that, different data sets of soil quality indicators have been proposed to discriminate between soil textural classes for different agricultural management systems and a variety of crops [9], [10], [11], [12], [13]. Besides the well known chemical and physical parameters used as soil quality indicators, such as soil organic matter and soil structure, there is still no consensus about biological soil indicators of sustainable agricultural systems. The massive adoption of no-till practices in extensive agriculture in Argentina gave rise to many situations, in which improvement of crop yield could not be associated to established quality indicators, but to the history of the soil management, suggesting that additional biological parameters might be necessary to describe changes in soil quality.

By driving crucial soil processes, such as decomposition of organic materials and nutrient cycling, soil bacteria are key players in ecosystem functioning. The structure of the microbial community in soil, the distribution of microbial biomass and enzyme activity may be affected by several factors, such as farming systems [14], plant species [15], [16], [17], tree species, soil pH [18], soil type [19], tillage and crop rotation [20], [21], [22], [23], [24]. This is why it is also important to take into consideration microbiological indicators when evaluating soil quality [25]. Yet, understanding about the influence of bacterial community structure on soil quality, and inversely, revealing the effect of soil characteristics on the structuring of bacterial communities is still scarce. In particular, to our knowledge, no previous study has addressed these issues in the framework of crop productivity, as assessed by farmers’ records.

This work is a part of a larger effort to find microbiological indicators of sustainable agriculture in the framework of no-till farming. The project BIOSPAS (http://www.biospas.org/en) is a multidisciplinary research project, in which agricultural soil biology is approached by means of a polyphasic description [26]. We have considered three treatments, which were replicated as blocks in four agricultural sites located across a west-east transect in Argentine Central Pampas, having documented history of no-till management. Two treatments were related to contrasting agricultural management practices under no-till in terms of crop rotation, fertilization, pest management and agrochemical use, which in coincidence to farmers’ records of crop yield can be regarded as “Good no-till Agricultural Practices (GAP)” and “Poor no-till Agricultural Practices (PAP)”. The third treatment corresponded to non-cultivated soils nearby the agricultural sites, which were used as references for natural environments (NE).

Pyrosequencing of 16S RNA gene using barcoded sequence tags is a high-throughput technique that has the capability to provide sufficient coverage and sequence length to afford an extensive taxonomic description of soil biota, comparing multiple samples in a single run [27]. A highly variable region of the 16S rRNA gene is individually PCR-amplified using primers containing a barcoded sequence (pyrotag) that allows distinction between samples. Tagged amplicons are pooled at equimolar concentration and sequenced in a single reaction. Reads were later assigned to individual samples based on the barcode sequence. Subsequent comparison to databases allows the identification of bacterial taxa and their relative abundance within the community. Here, we used tag-encoded pyrosequencing to deeply sample the 16S rRNA gene from bacteria residing in soils corresponding to the three treatments at the four sites with the objective of finding out potential candidate bacterial species as indicators of agricultural management. As we have considered soils with varied characteristics, in terms of texture and organic matter, the identification of statistically based soil management-associated taxa can provide useful diagnostic tools for agricultural soil quality across the surveyed region.

## Materials and Methods

### Study Sites

The management and sites for this study were selected after a thoughtfully discussion between scientists and farmers participants of the BIOSPAS Project (www.biospas.org/en).

Whereas the sites selected may not fulfilled a rigorous definition of replicates, due to slight differences in management (historical crop sequence, years on no-till agriculture, were not the same), the experimental design privileged the perspective of farmers in terms of the relation between soil management and crop productivity. We have therefore followed a working definition of soil management, according to a set of definitions of Certified Agriculture by the Argentine No Till Farmers Association (AAPRESID, www.ac.org.ar/descargas/PyC_eng.pdf) and the Food and Agricultural Organization of the United Nations (FAO, www.fao.org/prods/GAP/index_en.htm).

Three treatments were defined (Table 1): 1) “Good no-till Agricultural Practices” (GAP): Sustainable agricultural management under no-till, subjected to intensive crop rotation (basically wheat/other winter crop/soybean/maize and sometimes including the use of cover crops, such as vicia/triticale), nutrient replacement, minimized agrochemical use (herbicides, insecticides and fungicides) and showing higher yield compared to PAP (Table 1); 2) “Poor no-till Agricultural Practices” (PAP): Non-sustainable agricultural management under no-till with high crop monoculture (soybean), low nutrient replacement, high agrochemical use (herbicides, insecticides and fungicides) and showing lower yields compared to GAP (Table 1); 3) “Natural Environment” (NE): As reference, natural grassland was selected in an area of approximately 1 hectare, close to the cultivated plots (less than 5 km), where no cultivation was practiced for (at least) the last 30 years.

**Table 1:** Description of the agricultural management and crop yield, averaged over the five years before the first sampling date, in June 2009 (2005–2009).

	Bengolea		Monte Buey		Pergamino		Viale	
	GAP	PAP	GAP	PAP	GAP	PAP	GAP	PAP
% no-tillage	100	80	100	100	100	100	100	100
Soybean/maize ratio^a^	1.5	4	0.67	4	1.5	5	1,5	4
% Winter with wheat^b^	60	40	60	20	40	0	40	20
% Winter cover crops^c^	20	0	40	0	0	0	20	0
Herbicide (L) used^d^	27.7	43.8	25.2	38.9	29.3	46.5	34.5	43.1
Soybean yield (kg.ha^−1^)	3067	2775	3167	2675	2933	2825	3000	1805
Maize yield (kg.ha^−1^)	10500	2700	12550	8000	9500	–e	7030	3450

a Number of soybean cycles to number of maize cycles over the last 5years.

b Percentage of winters that wheat was planted as a winter crop.

c Percentage of winters that a cover crop (*Vicia* sp., *Melilotus alba* or *Lolium perenne*) was planted. Cover crops were chemically burned before summer crops are planted.

d Calculated as liters of low-toxicity herbicides plus liters of moderate-toxicity herbicides weighted by two. Toxicity was defined according to EPA Toxicity Categories. Unit: total liters over 5 years.

e No maize was planted in the last 5 years.

Treatments were replicated 4 times (blocks) in agricultural fields located across a west-east transect in the most productive region in the Argentinean Pampas. Sites of soil sampling were near the following locations in Argentina: Bengolea at Córdoba Province (33° 01′ 31″ S; 63° 37′ 53″ W); Monte Buey at Córdoba Province (32° 58′ 14″ S; 62° 27′ 06″ W); Pergamino at Buenos Aires Province (33° 56′ 36″ S; 60° 33′ 57″ W); Viale at Entre Ríos Province (31° 52′ 59,6″ S; 59° 40′ 07″ W). See Table 2 for a description of soil characteristics.

**Table 2:** Soil characteristics according to site and agricultural management at the first sampling date, in June 2009.

	Bengolea			Monte Buey			Pergamino			Viale		
	NE	GAP	PAP	NE	GAP	PAP	NE	GAP	PAP	NE	GAP	PAP
Climate	Temperate Subhumid			Temperate Subhumid			Temperate Humid			Temperate Humid		
MAT^1^ (°C)	17			17			16			18		
MAP^2^ (mm yr^−1^)	870			910			1000			1160		
Altitude (m)	224	222	223	112	111	108	64	68	65	73	80	81
Slope (%)	0.5	0.75	0.5	0.01	0.5	0.2	0.25	0.5	0.5	0.75	0.75	0.2
Years of no-till		13	5		28	10		6	5		13	9
Soil classification	Entic Haplustoll			Typic Argiudoll			Typic Argiudoll			Argic Pelludert		
Texture	Sandy loam			Silt loam			Silt loam			Silty clay/Silty clay loam		
Carbon %	1.7	1.5	1.1	3.5	2.1	1.7	2.7	1.7	1.8	5	3.5	2.5
Nitrogen %	0.146	0.156	0.125	0.328	0.181	0.132	0.233	0.153	0.136	0.369	0.283	0.179
Extractable P (ppm)	44.3	53.1	17.8	296.5	126.5	20.6	10.5	18	11.9	20.2	40.4	41.8
pH	6.3	6.2	6.2	5.6	5.5	6.2	6.2	6	5.7	6.4	6.7	6.3
Moisture	10.58	7.96	6.32	25.47	21.87	18.03	22.83	22.03	12.73	17.8	25.3	18.2

1 Mat: Mean annual temperature.

2 MAP: mean annual precipitation.

### Sampling

Samples were taken in June 2009 (winter) as triplicate for each treatment-site in three 5 m^2^ sampling points separated at least 50 m from each other, taking care not to follow the sowing line in the field. Three additional samplings in the exact same locations were performed in February 2010, September 2010 and February 2011. Samplings were performed at private productive fields, which belong to any of the funders of this work. None of the sampling areas belong to a protected area or land. Permissions were obtained directly from the owners or responsible persons. At Bengolea and Monte Buey locations sampling was allowed by Jorge Romagnoli, from La Lucía SA, at Pergamino sampling was allowed by Gustavo Gonzalez Anta, from Rizobacter Argentina SA, sampling at Viale was allowed by Pedro Barbagelatta, member of Aapresid.

Each sample of the top 10 cm of mineral soil was collected as a composite of 16–20 randomly selected subsamples. Composite soil samples were homogenized in the field and transported to the laboratory at 4°C. Within 3 days after collection, samples were sieved through 2-mm mesh to remove roots and plant detritus. Soils were stored at −20°C until DNA extraction.

### Chemical and Physical Soil Properties

Soils were classified according to Soil Taxonomy and INTA (Instituto Nacional de Tecnología Agropecuaria, Argentina) soil map. The main chemical properties of soils were determined by standard methods on samples that were air-dried, crushed and passed through a 2-mm sieve after removal of plant residues. The pH was determined on mixtures of 1∶2.5 sample:water. The contents of organic matter as total organic carbon were measured by dry combustion using a LECO CR12 Carbon analyzer (LECO, St. Joseph, MI, USA). The total nitrogen contents in whole soils were obtained by the Kjeldahl method. Extractable phosphorus was determined by the method of Bray and Kurtz. Data is summarized in Table 2.

### DNA Extraction

Further homogenization was performed by careful grounding 10–15 g of each soil sample in a mortar before DNA extraction. DNA was extracted from 0.5 g of soil using FastDNA spin kit for soil extraction kit (Mpbio Inc), following the manufacturer’s instructions. In order to reduce the presence of humic substances that inhibited the subsequent PCR reaction, an additional purification step was performed on the DNA sample using polyvinyl polypyrrolidone (PVPP). Eluted DNA was stored at −20°C.

### Pyrosequencing

Barcoded pyrosequencing analysis was run on samples from the first sampling date of the BIOSPAS project, in June 2009. DNA samples were diluted to 10 ng/µL and 1.5 µL DNA aliquots of each sample were used for 50 µL PCR reactions. A fragment of the 16S rRNA gene of approximately 525 bp in length was amplified using bacterial primer 27F and universal primer 518R, both containing a unique 10-bp barcode sequence per sample to facilitate sorting of sequences from a single pyrosequencing run. PCR was conducted with 0.3 mM of each forward and reverse barcoded primer, 1.5 µl template DNA, 2X buffer reaction, 0.2 mM of dNTPs, 1 mM MgSO_4_ and 1U of Platinum *Pfx* DNA polymerase (Invitrogen). Samples were initially denatured at 94°C for 5 min, then amplified using 35 cycles of 94°C for 30s, 50°C for 30 s and 68°C for 30s. A final extension of 10 min at 68°C was added at the end of the program to ensure complete amplification of the target region. Each of the triplicate subsamples was amplified separately and later combined and used as a representative composite of each sample. Amplicons were gel purified using GFX PCR DNA and Gel Band Purification Kit (GE Healthcare), and sent to the Genome Project Division Macrogen Inc. Seoul, Republic of Korea to be run on a Roche Diagnostics (454 Life Science) GS-FLX instrument with Titanium chemistry.

### Sequence Data Analysis

Data were processed using MOTHUR v.1.22.2 following the Schloss SOP [28]. Briefly, the 10-bp barcode was examined in order to assign sequences to samples. Sequencing errors were reduced by implementation of the AmpliconNoise algorithm and low-quality sequences were removed (minimum length 200 bp, allowing 1 mismatch to the barcode, 2 mismatches to the primer, and homopolymers no longer than 8 bp). Sequences with ambiguous bases were eliminated as well, as their presence appear to be a strong indication of defective sequences [29]. The choice of these parameters for filtering follows the recommendation of Schloss et al. to reduce the error rate [30].

Chimera were removed with ‘chimera.uchime’ Mothur command. Sequences were aligned and classified against the SILVA bacterial SSU reference database v 102 [31]. Following the OTU approach [28], sequences from forward primers were clustered according the furthest neighbor-clustering algorithm.

Pyrosequencing raw reads were deposited in the NCBI Short-Read Archive under accession SRA057382. Sequence profile of processed sequences is shown in table S1. Raw and filtered reads per sample are shown in table S2.

Shared tables were created indicating the number of times an OTU appears in each sample. Venn diagrams were constructed with package gplots in R 2.10.1 (http://www.R-project.org/) from shared tables at the 0.05 distance level. Names and sequences from shared phylotypes were retrieved with scripts written in Python.

An indicator value analysis was performed for detecting statistically significant associations between taxa and soil management [32]. The indicator value combines the abundance of the OTU in the target group compared to other groups (specificity), with its relative frequency of occurrence in that particular group (fidelity). The value of the IndVal index was calculated using function IndVal in [R] package ‘labdsv’ (http://ecology.msu.montana.edu/labdsv/R/labdsv). Data were previously ANOVA filtered to reduce the number of tests and therefore increase the power to detect true differences. In order to perform multiple testing corrections, analysis of false-discovery rate (FDR) of 0.05 of significance were calculated for the complete set of p-values with qvalue.gui() in [R] package ‘qvalue’ (http://genomics.princeton.edu/storeylab/qvalue/). The FDR estimates the chance of reporting a false-positive result in all the significant results [33].

### Real-time PCR Quantification

Bacterial taxa were quantified using the taxon-specific 16S rRNA primers designed in this work. All primers were designed using the PRIMROSE software [34].

For bacteria within the GP1 group of the phylum Acidobacteria, two specific primers were designed using the sequences available at the Ribosomal Database Project (RDP) database (http://rdp.cme.msu.edu/probematch/search.jsp) and the 100 sequences of the selected OTU.

Primers for the genus *Rubellimicrobium* Rub290F (GAGAGGATGATCAGCAAC) and Rub547R (CGCGCTTTACGCCCAGTC) were designed using all the *Rubellimicrobium* sequences available in the RDP database. The specificity of the primers Gp1Ac650R (TTTCGCCACAGGTGTTCC) and SubGp1-143F (CGCATAACATCGCGAGGG) were initially checked by *in silico* analysis against RDP probe match (data set options: good and >1200 bp). The Gp1Ac650R primer matched with 92.4% of the sequences within the class Acidobacteria Gp1 (2268/2454), and with other 394 non-target bacteria. The SubGp1-143F primer matched with 99/100 sequences of the indicator group and only 3 sequences of non-target bacteria in the RDP database. More than 98% of the sequences in the RDP database (105/110) matched the primers combination Rub290F (GAGAGGATGATCAGCAAC) and Rub547R (CGCGCTTTACGCCCAGTC). Only 2 sequences of non-target bacteria in the RDP database matched with this primers combination.

To further test the specificity of the qPCR assays, clone libraries were constructed for each primer set using DNA extracted from GAP soil samples of Pergamino and Monte Buey for Acidobacteria GP1, and PAP soil samples of Bengolea and Monte Buey for *Rubellimicrobium*. Twenty four positive clones of each library (n  = 48 for each primer set) were sent to Macrogen Inc. for complete sequencing. Sequences were assigned to taxonomic groups using the RDP classifier program. 100% of the cloned amplicons that could be identified belonged to the correct target groups.

Quantification was based on the increasing fluorescence intensity of the SYBR Green dye during amplification. The qPCR assay was carried out in a 20µL reaction volume containing the SYBR green PCR Master Mix (Applied Biosystems, UK), 0.5 µM of each primer, 0.25 µg/µL of BSA and 10 ng of soil DNA. Primer annealing temperature was optimized for PCR specificity in temperature-gradient PCR assays, utilizing the DNA Engine Opticon 2 System (MJ Research, USA). Optimal conditions for PCR were defined as 10 minutes at 94°C, 35 cycles of 94°C for 30 seconds, 59°C for 20 seconds and 72°C for 30 seconds, for both sets of primers. Standard curves were obtained using at least five ten-fold serial dilutions of a known amount of PCR amplicon mixtures as templates, purified through QIAquick PCR purification columns. Controls with no DNA templates gave null or negligible values.

### Statistical Analysis

Patterns of similarity between samples were investigated using Correspondence Analysis (CA) on the relative abundances of OTUs_0.05_. Due to the fact that the number of sequences obtained for NE of Monte Buey was markedly lower than those obtained for all other samples, this sample was excluded from this type of analysis.

Correlation between relative abundances of all significant indicator OTUs_0.05_ and soil environmental gradients was assessed using Canonical Correspondence Analysis (CCA). The model used to explain variability included moisture content, total nitrogen content, total carbon content, ratio of total carbon to total nitrogen content and pH. Abiotic variables were standardized by subtracting the mean and dividing by the standard deviation (z-score standardization), making quantitative variables dimensionless. Significance was assessed using permutation tests. Multivariate analyses were performed in [R] package ‘vegan’.

The effect of site and management on the number of copies of 16S rRNA genes for GP1A (the indicator group within the GP1 of the Acidobacteria) and *Rubellimicrobium*, was determined for each sampling date by mixed models. Treatment and seasons (summer, winter) were considered as fixed factors, whereas year, site, and subsample were included in the random structure. For this analysis, data were log transformed to achieve normal distribution of residues. The mean number of 16S rRNA gene copies of each taxon was compared for the effect of treatment by orthogonal contrasts.

Management indicator value was defined as the logarithm of the ratio of the normalized abundance (i.e. fold-change relative to a non-cultivated soil) of the GP1A and the normalized abundance of *Rubellimicrobium* template. Two-tailed one-sample *t*-tests were performed on mean management indicator values (n = 48 for each type of management) to test the null hypothesis that the mean was equal to 0, and 95% confidence intervals were calculated. Statistical analysis was carried out with InfoStat Plus version 2011 (http://www.infostat.com.ar).

## Results

### Soil Quality and Productivity According to Sites and Agricultural Practices

The information on the agricultural management and crop yields of the different sites under study are summarized in Table 1. Before the first sampling, all sites had been managed under no-till for at least the preceding five years, with the exception of a single year (2004/2005) in Bengolea, where the PAP site was chisel-plowed. In the four localities, GAP had in average a 62% higher proportion of maize in the crop rotation than the PAP. GAP had in the last five years 50% of the winters with crop, whereas PAP sites had only 20%. In addition, cover crops had been implanted in winter in three of the four GAP localities. Management also differed in terms of the amount of herbicides used, as soils under PAP had used 36% more herbicides than GAP during the previous five years. Soybean yield had been in average 24.7% higher in GAP than in PAP, whereas maize yield had been 149.9% higher in GAP.

Soil chemical and physical properties of the studied sites are presented in Table 2. There is a difference in soil texture among localities, with increasing clay and decreasing sand content from West (Bengolea) to East (Viale). Values of soil organic matter follow the relation NE>GAP>PAP at the different localities, except in Pergamino where the Good no-till Agricultural Practices (GAP) and the Poor no-till Agricultural Practices (PAP) showed similar values. Soil N content also followed the same pattern, with the exception of Bengolea, where GAP had higher values than NE. No clear association was observed between values of extractable P and soil type or management. The pH, which ranged from 5.5 to 6.7, did not appear to correlate with soil type or soil management. A more detailed analysis, comparing physical and chemical soil properties of the different agricultural management under study, exceeds the purpose of this paper and will be presented elsewhere (Duval et al, unpublished).

### Bacterial Community Structure

The structure of bacterial communities related to the agricultural management practices was obtained from the massive parallel sequencing data of the 12 samples, i.e. three management scenarios over the four locations. A total of 210579 sequences with an average read length of 284 bp, were obtained after trimming, sorting, and quality control of the pyrosequencing data (table S2). 80% of these sequences were classified to a known phylum in the domain Bacteria (Fig. S1).

We examined OTU distribution across the pyrosequencing-based data sets. Using 3% sequence variation criterion, the patterns of the rarefaction curves were roughly comparable in all samples and none of the curves reached a plateau (Fig. S2). Therefore it was not possible to establish a trend in the differences of richness as a function of either geographical location or soil management. Since, despite quality filtering, pyrosequencing has a large intrinsic error that could lead to overestimation of rare phylotypes, further estimation of bacterial richness was not attempted.

### Taxa Overlap between Soil Samples

**Figure 1:**
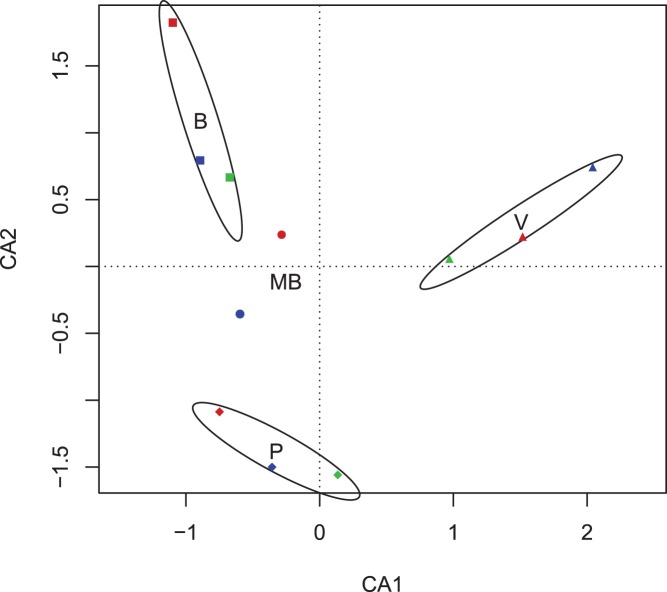
Ordination diagram from the Correspondence Analysis of the relative abundances for taxa defined at 0.05 distance. The 2-D CA diagram account for 29% of inertia. Locations of soils are indicated by squares (Bengolea, B), circles (Monte Buey, MB), diamond (Pergamino, P) and triangles (Viale, V). Colors indicate soil management type: Poor no-till Agricultural Practices in red, Good no-till Agricultural Practices in blue and Natural Environment in green. Standard error ellipses show 95% confidence areas.

Correspondence Analysis (CA) was applied to the data set of relative abundances for taxa defined at 0.05 distance (Fig. 1). Axes 1 and 2 account for 28.8% of the total inertia (16.4% and 12.4%, for axes I and II, respectively). Fitting of environmental factors to ordination indicate that samples were distributed according geographical location (site) with p = 0.001.

**Figure 2:**
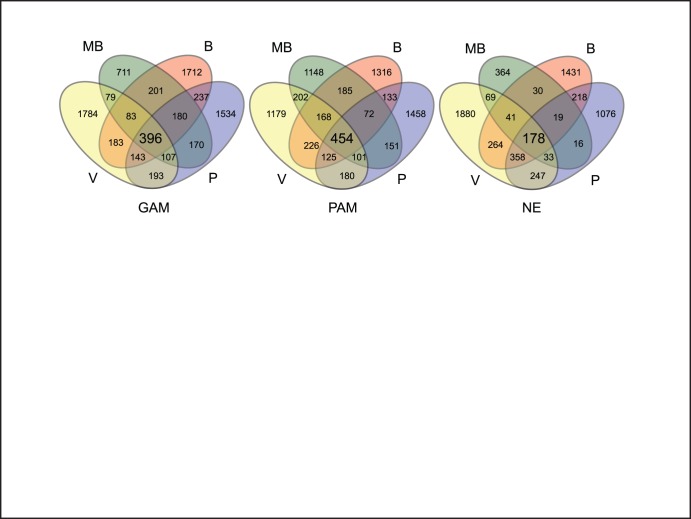
Venn diagram of the overlap of OTUs from the different soil management practices at four geographical locations. The numbers of overlapping tag sequences are indicated in the graph. Management practices are indicated at the bottom of each diagram: GAP: Good no-till Agricultural Practices, PAP: Poor no-till Agricultural Practices; NE: Natural Environment. Location labels are indicated with B (Bengolea), MB (Monte Buey), P (Pergamino) and V (Viale).

The majority of OTUs were unique to the samples in which they were found. The Venn diagrams in Fig. 2 show the number of shared OTUs among the different sample types. When analyzed by soil management, 254 OTUs were common to GAP and PAP samples across all sites (Table S3). Considering only sequences that were found in one type of management, but absent in the other, GAP and PAP samples had respectively 142 and 200 OTUs in common among the four sites, which corresponded to around 1.0% and 1.4% of the total number of sequences (Tables S4 and S5).

Considered by geographical location, the number of common OTUs was around 11% of the total OTUs identified in each location. In these cases, the overlap for the three samples in each sampling location was similar to the numbers of OTUs shared by the pair GAP and NE and the pair GAP and PAP, which in turn were consistently higher than the overlap of OTUs shared by NE and PAP (Fig. S3). We did not detect any OTU that was common to NE and PAP, but absent in GAP, even when the sample of NE from Monte Buey, which had less sequences, was excluded from the analysis. This finding disputes the possibility that the overlaps between groups of samples were due to chance.

### Indicator Taxa of Agricultural Management

Table 3 shows the result of the IndVal analysis for indicators containing more than 20 sequences, which identified four significant indicators of GAP and five significant indicators of PAP.

**Table 3:** Results of indicator species analysis.

	Size	IndVal	Freq	p value	q value	Phylogenetic affiliation
GAP	100	0.86	8	0.028	0.041	Acidobacteria_Gp1
PAP	76	0.78	9	0.032	0.041	*Rubellimicrobium*
GAP	55	0.91	7	0.050	0.041	Alphaproteobacteria
PAP	34	0.85	8	0.037	0.041	*Micromonosporaceae*
PAP	28	0.75	8	0.043	0.041	Acidobacteria_Gp16
GAP	26	0.85	7	0.014	0.041	Unclassified bacteria
GAP	23	0.83	6	0.044	0.041	Unclassified bacteria
PAP	20	1.00	4	0.009	0.041	Unclassified bacteria
PAP	20	0.80	7	0.038	0.041	Actinomycetales

For each of the taxa, we indicate the total number of sequences corresponding to the OTU that represents the specific groups of samples (size), the Indicator Value index (IndVal), the number of samples that contain the taxon (Freq), the statistical significance of the association (p-value), the chance of reporting a false-positive result (q-value), and the lowest taxonomic rank assigned with a bootstrap confidence greater than 80%. Agricultural managements GAP and PAP are defined in the main text. Results were sorted according to Size. Only OTUs containing 20 or more sequences are shown in this table. See Table S6 for a complete list of significant indicators with IndVal values ≥0.75.

**Figure 3:**
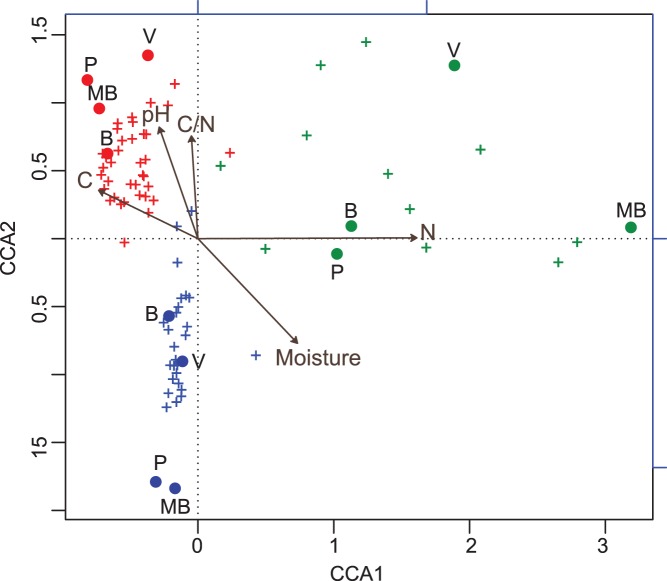
Ordination diagram from Canonical Correspondence Analysis of bacterial taxa identified as indicators using IndVal. Only OTUs identified with IndVal values higher than 0.75 were used in this analysis (Table S6). The 2-D ordination diagram CCA accounts for 66% of inertia. Samples are indicated by circles and site labels. OTUs are indicated by crosses, names are omitted. Arrows for quantitative variables show the direction of increase of each variable, and the length of the arrow indicates the degree of correlation with the ordination axes. Colors indicate soil management type: Poor no-till Agricultural Practices in red, Good no-till Agricultural Practices in blue; Natural Environment in green. Location labels are indicated with: B (Bengolea), MB (Monte Buey), P (Pergamino) and V (Viale).

Canonical Correspondence analysis (CCA) was applied on the bacterial taxa identified as indicators using IndVal to study the association of physico-chemical soil properties to sites and taxa (Fig. 3). Bacterial taxa clustered into three well-separated groups associated with the different soil management practices despite the different geographic origin of the data. The first ordination axis was strongly correlated to total nitrogen content (0.96, p<0.05), meaning the bacterial indicators of natural environments were associated with higher than average nitrogen content. The second canonical axis correlated in descending order to the pH (0.49), moisture (−0.47), carbon to nitrogen ratio (0.45) and total carbon (0.22). Separation between management indicators was influenced by the second canonical axis. Indicators of PAP are located in the positive quadrant. i.e. they occur at sites with higher than average pH, and carbon to nitrogen ratio, and lower than average values of moisture. Inversely, GAP indicators were associated with higher moisture content, lower pH and lower carbon to nitrogen ratio.

Although significant after the application of the false discovery rate, most of the indicators had low abundance across the samples. To obtain meaningful quantitative results, we analyzed only significant indicator containing at least 75 sequences. As a result, we selected single the list’s top indicators for GAP and PAP samples respectively, belonging to a taxa within the Acidobacteria Group 1 (GP1A), and to *Rubellimicrobium*, a genus of the order *Rhodobacterales* of the class Alphaproteobacteria (Table 3). Although this threshold can be considered somewhat arbitrary, it was selected on the basis of the fact that those taxa were represented more evenly across all studied locations.

**Figure 4:**
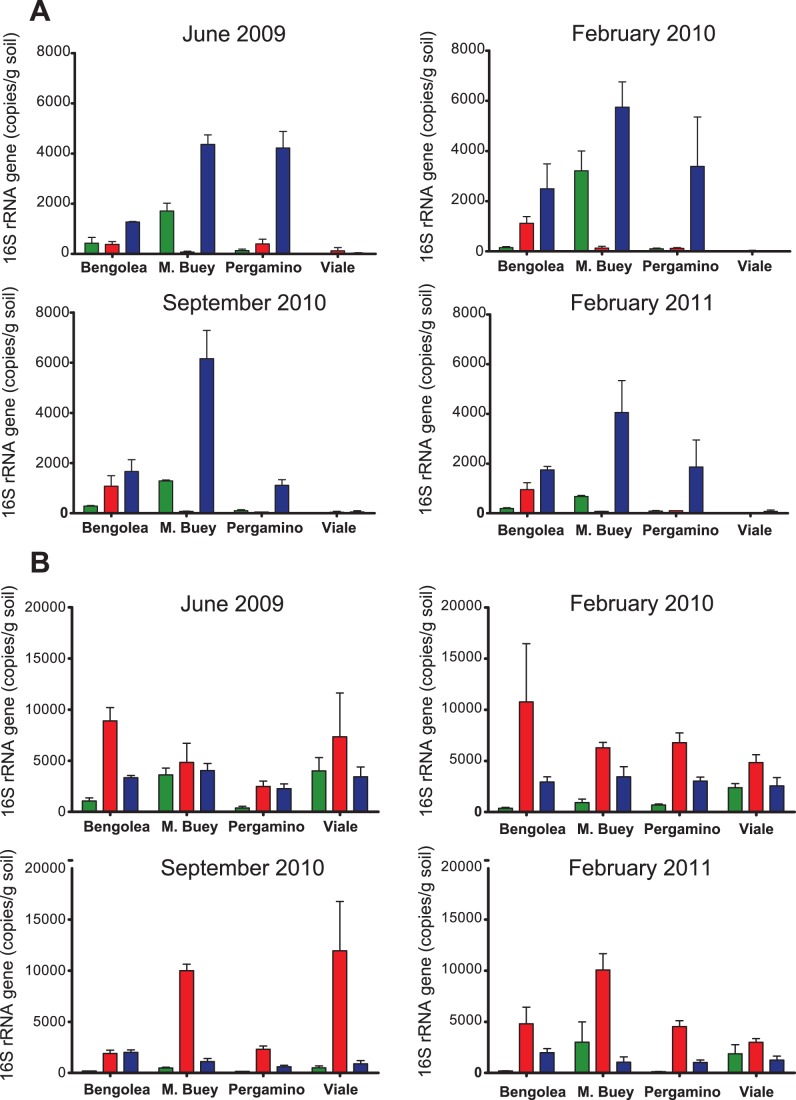
Quantitative phylogenetic group abundance of the OTUs targeted with a set of primers specific for Acidobacteria GP1A (panel A) and *Rubellimicrobium* genus (panel B). Each soil sample subjected to the indicated management in the four geographical locations was sampled at the date showed in the boxes. Bars correspond to the average qPCR data of three independent samples. Colors indicate soil management type: Poor no-till Agricultural Practices in red, Good no-till Agricultural Practices in blue, and Natural Environment in green. Error bars are standard error.

PCR primers were designed to target the sequences detected in the pyrosequence data set of these selected groups. Cloning and sequencing of the PCR products derived from the primer specificity tests confirmed the specificity of these primers (see M&M). Using these newly designed primers, quantitative PCR was conducted to validate the results of the sequence analysis. Primer sets of both Acidobacteria GP1A and the genus *Rubellimicrobium* were calibrated using known concentrations of clones of PCR amplified 16S rRNA genes from the respective controls. The quantification of a set of Acidobacteria GP1A and of the genus *Rubellimicrobium*, performed over samples from two successive winter-summer seasons (June 2009 to February 2011) are shown respectively in Fig. 4 A and B. Quantitative PCR data revealed that the abundance of both taxa were significantly different among managements (mixed models, p<0.0098 for GP1A and p<0.0001 for *Rubellimicrobium*). Post-hoc contrasts indicated that the number of copies of 16S rRNA genes targeted with GP1A-specific primers were statistically higher in samples of soils defined as GAP (p = 0.005), compared to poorly managed soils. In opposition, the number of copies of 16S rRNA genes targeted with *Rubellimicrobium*-specific set of primers were statistically higher in samples of soils defined as PAP (p = 0.004).

**Figure 5:**
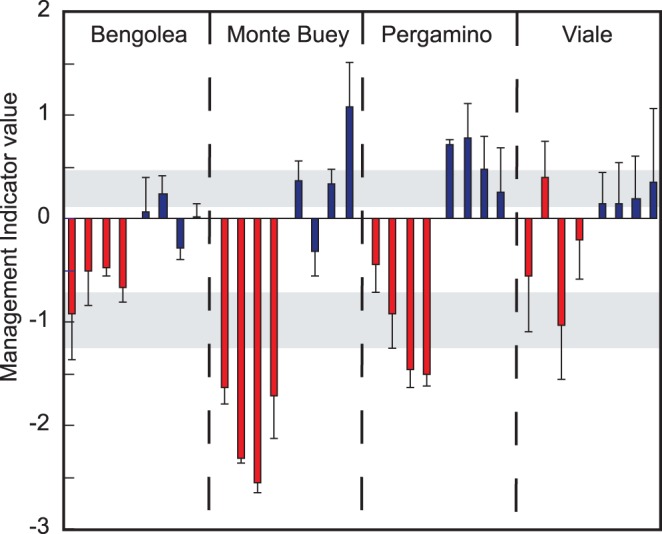
Indicator values for samples of soils under different agricultural management. The geographic sites are indicated in the box. In each site bars are ordered from left to right to the successive sampling dates: June 2009, February 2010, September 2010 and February 2011. PAP, Poor no-till Agricultural Practices (red) and GAP, Good no-till Agricultural Practices (blue). Shadow areas are 95% confidence intervals of indicator of GAP (0.24, 0.71) and PAP (−1.31, −0.41). Error bars are standard error.

The observation that the GP1A-specific primers target sequences that increase in GAP samples and that the *Rubellimicrobium*-specific primers target sequences that increase in PAP samples, prompted us to evaluate their combined use as potential indicator of agricultural management under no-till regime in the Pampa Region. For that purpose, we calculated the ratio of the normalized abundance (i.e. fold-change relative to a non-cultivated soil in the same geographical location) of the GP1A and the normalized abundance of *Rubellimicrobium* template. By definition, this value is equal to one for NE samples. Log transformation was applied to achieve normal distribution. The resulting indicator value will take therefore a value of zero for NE samples. For all other samples, the sign of the indicator will depend on whether the GP1A and *Rubellimicrobium* abundances increase or decrease relative to NE. The results are indicated in Fig. 5. Despite the high variability of the data, likely due in part to the heterogeneous distribution of bacteria in subsamples within each given sample, the mean of the value calculated for all GAP samples across the four sites and four sampling dates (n = 48) was significantly higher than 0 (p = 0.0018), whereas the mean of the value in all PAP samples was significantly lower than 0 (p<0.001).

## Discussion

The main hypothesis tested in this study was that the relative abundance of selected soil bacterial taxa could be used as indicator of the impact of agronomic management at a regional scale. Based on massive parallel sequencing and quantitative PCR, we have found that the combined use of the abundance of two bacterial taxa could potentially fulfill this task. The bacteria, belonging to Acidobacteria Group 1, and to the genus *Rubellimicrobium* of the Alphaproteobacteria, were augmented in soils under no-till crop production, managed with sustainable and non-sustainable practices, respectively. What makes our finding more compelling is that the taxa that appeared to be specific of soil management, were present in soils with different physical properties (Table 1) with various crop sequences (Table 2), suggesting that the physiology of these group of bacteria might be affected by nutrient and carbon shifts, and probably different soil microstructure, produced by the different crop rotation practices: intense crop rotation vs. monoculture practice, the most important characteristic differentiating soil managements, with consistent different yields.

Agricultural soil activities should sustain crop productivity while preserving soil environmental quality. After several years of no-till agriculture and the widespread practice of monoculture, farmers have realized the impact of management on soil quality, which ultimately impacted on crop productivity. This has led to a working definition of “Good no-till Agricultural Practices” (GAP) and “Poor no-till Agricultural Practices” (PAP), according to criteria based on yield, crop rotation, fertilization, pest management and agrochemical use (http://www.ac.org.ar/index_e.asp). In this context, indicators of soil quality are essential tools to evaluate the impact of management on the soil ecosystem. Physical properties, such as soil structure, water storage capacity and soil aeration, as well as soil chemical characteristics are currently used as indicators of soil health. In addition, microbial properties are increasingly regarded as more sensitive and consistent indicators than biochemical parameters for monitoring the effect of management on soil quality [35]. This is because bacteria are in intimate contact with the soil microenvironment. Microorganisms can be directly affected by some toxic effect, or indirectly, e.g. by changes in the availability of substrates, and therefore the energy available for growth [36].

Previous studies have related the effect of different management practices on the diversity and stability of microbial communities and the abundance of individual taxa, in carefully designed experimental plots, using PLFA profiling [37], [38], phenotypic fingerprinting [23], ribosomal fingerprinting [39], and pyrosequencing [20], [24]. However, we are not aware of a study that looked for indicators of soil management in the large spatial and temporal scale of agricultural practices tested in productive fields. This task is particularly challenging, as most bacteria present discernible biogeographical patterns, even within a given habitat type [40]. Accordingly, in a large-scale investigation on the relative importance of various soil factors and land-use regimes on soilborne microbial community composition, it was found that the main differences in the bacterial communities were related to soil factors [41].

In our samples we have observed that bacterial communities as a whole appeared indeed to be structured chiefly by geographical proximity, meaning that differences in composition are due mainly to soil characteristics at the landscape scale [42]. Nevertheless, it was particularly interesting that the distribution of certain bacterial populations was clearly shaped by factors determined by soil management as well, opening the window to find bacterial indicators of soil status across a broad spatial scale [24]. The numbers of OTUs, which were found to be common to the four soil locations subjected to similar management practice, was relatively large. We deem unlikely that this overlap was the outcome of chance alone, as for each type of management the number of OTUs shared by any three of the four geographical locations was lower than the number of OTUs common to the four soil samples (Fig. 2). Even more striking is the observation of bacterial groups that can be associated with soil management in agricultural soils with dissimilar characteristics across a relatively wide regional scale. These data are consistent with both genomic and environmental perspectives suggesting the existence of ecological coherence of bacterial at different taxonomic ranks [43].

The set of indicator taxa were used to evaluate the correlation of their abundances with soil characteristics across sites and management. The ordination illustrated how indicator taxa were responsive to soil management practices. GAP indicators were associated with higher moisture content, and lower carbon to nitrogen ratio and lower pH. Slight changes in pH might have been caused by acidifying reactions (e.g. nitrification). Inversely, the occurrence of PAP indicators at sites was associated with higher than average carbon to nitrogen ratio, i.e. under conditions in which nitrogen becomes a limiting factor.

The phylum Acidobacteria ranked third in abundance in each of the twelve soil samples examined in this study. Acidobacteria constituted an average of 20% of soil bacterial taxa in 16S rRNA gene libraries, according to a published meta-analysis [44] and more recent analysis of agricultural soils indicated that three subgroups (GP4, GP6 and GP1) situate among the five most abundant genera in soils [24], [45]. Although the phylum Acidobacteria it has been frequently associated with low nutrient availability [46], its wide global distribution and high diversity led to the proposition that its members are involved in a broad range of metabolic pathways [47]. Several findings point to the fact that not all subdivisions within the phylum Acidobacteria share the same traits. Examples of these are the occurrence of numerically dominant as well as metabolically active Acidobacteria in rhizospheric soil [45], the lineage-dependent variations in relative abundance within a clay fraction of soil versus bulk soil [48] and the differences in the pH preferences for growth [44]. Interestingly, Mummey et al found that Acidobacteria were poorly represented in the inner fraction of aggregates [49], but were more abundant in soil macroaggregates and the outer fractions of microaggregates, i.e. in coarse pores, where they are supposed to have high turnover rates because of the effect of predation and desiccation events, and due to the transiently high oxygen and nutrient availability [50].

It is therefore not entirely surprising that a subgroup of the Acidobacteria group 1 emerges as a potential bacterial candidate for agronomic practices in soils managed under no till regime, in which carbon conservation and stability of macroagregates are enhanced (Morras et al, personal communication). Unraveling specific details about the ecology of this particular lineage of Acidobacteria through cultivation [51] and genomic studies [52] are needed to gain a better understanding of its involvement in soil processes.

Neither is the natural habitat of Rubellimicrobia currently well characterized. To date, four species of the genus *Rubellimicrobium* had been described. One thermophilic species, *R. thermophilum*, which was isolated from slime deposits on paper machines and a pulp dryer [53] and three mesophilic species, two of which have been isolated from soils: *R. mesophilum* [54] and *R. roseum* [55], and *R. aerolatum*, which was isolated from air samples [56]. It is worth noting that fatty acids profiles of the same soil samples analyzed in this work, show in all PAP treatments that fatty acid C_18∶1_ω7c is significantly augmented (Ferrari and Wall, unpublished). This is relevant because C_18∶1_ ω7c is one of the major membrane fatty acids in most of the isolates belonging to the genus *Rubellimicrobium* [54], [55], [56]. Given the limited physiological and ecological information available on the genus *Rubellimicrobium*, it would be too speculative to suggest for it any indicator function in soils at the present time. Nevertheless, it is interesting to note that this genus appeared to respond to the use of the soil in a recent study of the impact of long-term agriculture on desert soil, in which it was shown that *Rubellimicrobium* was among the extremophilic bacterial groups that disappeared from soil after agricultural use [57]. Efforts to isolate *Rubellimicrobium* strains from the soils surveyed in the present study are currently under way in our laboratory, in order to perform a thorough physiological characterization.

The results of this study provide relevant information about the distribution of several groups of numerically abundant taxa in agricultural soils. It was also demonstrated that different taxa of bacteria respond differentially to geographical constraints and contemporary disturbances in no-till agriculture systems, highlighting the potential of high-resolution molecular tools to identify bacterial groups that may serve as potential indicator that might be used to assess the sustainability of agricultural soil management and to monitor trends in soil condition over time.

We note that the selection of indicator species based solely on the frequency of occurrence does not permit conclusions about the processes in which they are involved. In this regard, knowledge on their habitat specialization would be important, as this factor is not likely to be influenced by natural variations in environmental conditions [58]. However, considering the scarcity of data regarding the habitat preferences, physiology and in situ activity of Acidobacteria GP1 and *Rubellimicrobium*, a mechanistic link between the factors driving the relative distribution of these taxa and the different soil management is currently not feasible. Thus, although we have initially developed the indicator on a purely phenomenological basis, the understanding of the underlying ecological selection for both groups of taxa depending on the soil management remains a crucial goal for future studies.

Meanwhile, the proposed marker appears to fulfill several of the criteria required for appropriate ecological indicators. It is easily measured, it is sensitive to soil management actions and is integrative, i.e. it provides adequate coverage across a relatively wide range of ecological variables, e.g. soils types, climate, crop sequence, etc. [59]. Based on the data presented here, appropriate tests for simple monitoring can be elaborated to further validate if the proposed candidate biological indicator can be integrated into a minimum dataset, to allow measuring the impact of management practices under no-till at the regional scale.

## Supporting Information

Figure S1 Complete set of sequences classified at phylum level against SILVA bacterial SSU refer- ence database v.102 by Bayesian method, with a confidence cutoff of 80% using classify.seqs command in Mothur.
(EPS)

Figure S2 Rarefaction analysis of pyrosequencing tags of the 16S rRNA gene in soils subjected at different agricultural practoces in the four geographic locations. Blue: Good no-till agricultural practices. Red: Poor no-till agricultural practices, Green: Natural environments.
(EPS)

Figure S3 Venn diagram of the overlap of OTUs from the different geographical locations subjected to different soil management practices. The numbers of overlapping tag sequences are indicated in the graph. Locations are indicated at the bottom of each diagram. Management practices are indicated with GAP: Good no-till Agricultural Practices, PAP: Poor no-till Agricultural Practices; NE: Natural Environment.
(EPS)

Table S1 Summary of processed 454-sequencing reads.
(DOCX)

Table S2 Filtered and raw (in parenthesis) reads of 454 Pyrosequencing per sample.
(DOCX)

Table S3 List of OTUs common to good no-till agricultural practices (GAP) and poor no-till agricultural practices (PAP) in the four locations. Sequences were assigned to taxonomic groups using the RDP classifier (http://rdp.cme.msu.edu/classifier/classifier.jsp). OTUs were sorted by the total number of sequences in the complete data set.
(DOCX)

Table S4 List of OTUs only common to good no-till agricultural practices (GAP) in the four locations. Sequences were assigned to taxonomic groups using the RDP classifier (http://rdp.cme.msu.edu/classifier/classifier.jsp). OTUs were sorted by the total number of sequences in the complete data set.
(DOCX)

Table S5 List of OTUs common only to poor no-till agricultural practices (PAP) in the four locations. Sequences were assigned to taxonomic groups using the RDP classifier (http://rdp.cme.msu.edu/classifier/classifier.jsp). OTUs were sorted by the total number of sequences in the complete data set.
(DOCX)

Table S6 Results of indicator species analysis. For each of the taxa, we indicate the Indicator Value index (IndVal), the number of samples that contain the taxon (Freq), the statistical significance of the association (p-value), the total number of sequences corresponding to the OTU (size), and the lowest taxonomic rank assigned with a bootstrap confidence indicated in parenthesis. Agricultural managements GAP and PAP an NE are defined in the main text. Only OTUs with IndVal higher than 0.75 are shown.
(DOCX)
