# Supplementary material for: Bacterial Indicator of Agricultural Management for Soil under No-Till Crop Production
Source: PLoS One. 2012 Nov 30;7(11):e51075. doi: 10.1371/journal.pone.0051075 (PMC3511350; doi:10.1371/journal.pone.0051075)
Supplement: Table S1 — Summary of processed 454-sequencing reads. (DOCX) [file pone.0051075.s004.docx]

Table S1- Summary of processed 454-sequencing reads

**Nbases N^1^ Polymer^2^ NumSeqs**

Minimum: 243 0 3 1

2.5%-tile: 278 0 4 2867

25%-tile: 295 0 4 28668

Median: 307 0 5 57336

75%-tile: 315 0 5 86003

97.5%-tile: 325 0 6 111804

Maximum: 346 0 8 114670

Mean: 304.646 0 4.65

# of unique seqs: 44158

total # of seqs: 114670

^1^ N: Number of ambiguous bases in sequence reads

^2^ Polymer: length of homopolymer runs in sequence reads
