# Supplementary material for: Bacterial Indicator of Agricultural Management for Soil under No-Till Crop Production
Source: PLoS One. 2012 Nov 30;7(11):e51075. doi: 10.1371/journal.pone.0051075 (PMC3511350; doi:10.1371/journal.pone.0051075)
Supplement: Table S2 — Filtered and raw (in parenthesis) reads of 454 Pyrosequencing per sample. (DOCX) [file pone.0051075.s005.docx]

Table S2 - Filtered and raw (in parenthesis) reads of 454 Pyrosequencing per sample

| **Bengolea** | | | **Monte Buey** | | | **Pergamino** | | | **Viale** | | |
| --- | --- | --- | --- | --- | --- | --- | --- | --- | --- | --- | --- |
| GAP | PAP | NE | GAP | PAP | NE | GAP | PAP | NE | GAP | PAP | NE |
| 19602 | 15121 | 18049 | 16565 | 19945 | 2218 | 22309 | 16855 | 16162 | 22056 | 18787 | 22910 |
| (41091) | (29892) | (34958) | (33983) | (36922) | (4099) | (43297) | (32078) | (30698) | (41617) | (37298) | (41414) |

GAP: Good no-till Agricultural Practices

PAP: Poor no-till Agricultural Practices

NE: Natural Environments
