# Supplementary material for: Bacterial Indicator of Agricultural Management for Soil under No-Till Crop Production
Source: PLoS One. 2012 Nov 30;7(11):e51075. doi: 10.1371/journal.pone.0051075 (PMC3511350; doi:10.1371/journal.pone.0051075)
Supplement: Table S3 — List of OTUs common to good no-till agricultural practices (GAP) and poor no-till agricultural practices (PAP) in the four locations. Sequences were assigned to taxonomic groups using the RDP classifier (http://rdp.cme.msu.edu/classifier/classifier.jsp). OTUs were sorted by the total number of sequences in the complete data set. (DOCX) [file pone.0051075.s006.docx]

Table S3: List of OTUs common to good no-till agricultural practices (GAP) and poor no-till agricultural practices (PAP) in the four locations. Sequences were assigned to taxonomic groups using the RDP classiﬁer (<http://rdp.cme.msu.edu/classifier/classifier.jsp>). OTUs were sorted by the total number of sequences in the complete data set.

| **ID** | **Size** | **Phylum** | **Class** | **Order** | **Family** | **Genus** |
| --- | --- | --- | --- | --- | --- | --- |
| 1 | 4516 | Proteobacteria(100) | Alphaproteobacteria(100) | Rhizobiales(100) | Bradyrhizobiaceae(100) | unclassified |
| 19 | 3360 | Acidobacteria(100) | Acidobacteria_Gp4(100) | unclassified(100) | unclassified(100) | unclassified(100) |
| 2 | 2507 | Firmicutes(100) | Bacilli(100) | Bacillales(100) | Bacillaceae(100) | Bacillus(97) |
| 35 | 1548 | Proteobacteria(100) | Alphaproteobacteria(100) | Rhizobiales(100) | unclassified | unclassified |
| 17 | 1161 | Proteobacteria(100) | Alphaproteobacteria(100) | Rhizobiales(100) | unclassified | unclassified |
| 28 | 992 | Proteobacteria(100) | unclassified | unclassified | unclassified | unclassified |
| 83 | 934 | unclassified(98) | unclassified(98) | unclassified(98) | unclassified(98) | unclassified(98) |
| 4 | 933 | Proteobacteria(100) | Alphaproteobacteria(100) | Rhizobiales(100) | unclassified(100) | unclassified(100) |
| 25 | 868 | Actinobacteria(100) | Actinobacteria(100) | Actinomycetales(100) | Nocardioidaceae(99) | Marmoricola(87) |
| 20 | 723 | Proteobacteria(100) | Alphaproteobacteria(100) | Rhizobiales(100) | unclassified(100) | unclassified(100) |
| 56 | 679 | Proteobacteria(100) | Betaproteobacteria(99) | unclassified | unclassified | unclassified |
| 15 | 642 | Proteobacteria(99) | unclassified | unclassified | unclassified | unclassified |
| 24 | 640 | Actinobacteria(100) | Actinobacteria(100) | Solirubrobacterales(95) | unclassified(95) | unclassified(95) |
| 311 | 586 | Actinobacteria(100) | Actinobacteria(100) | Actinomycetales(100) | Micromonosporaceae(100) | unclassified(90) |
| 42 | 577 | Proteobacteria(100) | Alphaproteobacteria(100) | Sphingomonadales(100) | Sphingomonadaceae(100) | unclassified(98) |
| 164 | 574 | Proteobacteria(100) | Betaproteobacteria(100) | Burkholderiales(100) | Burkholderiales_inc. sedis(84) | unclassified |
| 9 | 565 | Proteobacteria(100) | Alphaproteobacteria(100) | Rhizobiales(100) | unclassified | unclassified |
| 6 | 507 | Proteobacteria(100) | Alphaproteobacteria(100) | Rhodospirillales(99) | unclassified(99) | unclassified(99) |
| 98 | 470 | Proteobacteria(100) | Betaproteobacteria(100) | Burkholderiales(100) | Comamonadaceae(100) | unclassified(87) |
| 79 | 453 | unclassified(100) | unclassified(100) | unclassified(100) | unclassified(100) | unclassified(100) |
| 199 | 448 | Chloroflexi(100) | Anaerolineae(100) | Anaerolineales(100) | Anaerolineaceae(100) | unclassified(100) |
| 466 | 430 | unclassified(100) | unclassified(100) | unclassified(100) | unclassified(100) | unclassified(100) |
| 102 | 424 | Proteobacteria(100) | Gammaproteobacteria(100) | Xanthomonadales(100) | Sinobacteraceae(100) | Steroidobacter(100) |
| 33 | 419 | Acidobacteria(100) | Acidobacteria_Gp1(100) | unclassified(100) | unclassified(100) | unclassified(100) |
| 37 | 413 | Proteobacteria(100) | Alphaproteobacteria(100) | Sphingomonadales(100) | Sphingomonadaceae(100) | unclassified |
| 21 | 407 | Acidobacteria(100) | Acidobacteria_Gp4(100) | unclassified(100) | unclassified(100) | unclassified(100) |
| 18 | 405 | Actinobacteria(100) | Actinobacteria(100) | Solirubrobacterales(100) | unclassified(100) | unclassified(100) |
| 68 | 405 | Actinobacteria(100) | Actinobacteria(100) | Actinomycetales(100) | Mycobacteriaceae(99) | Mycobacterium(99) |
| 54 | 381 | Proteobacteria(100) | unclassified | unclassified | unclassified | unclassified |
| 31 | 379 | unclassified(100) | unclassified(100) | unclassified(100) | unclassified(100) | unclassified(100) |
| 258 | 370 | Firmicutes(100) | Bacilli(100) | Bacillales(100) | Bacillaceae(100) | Bacillus(97) |
| 555 | 369 | Acidobacteria(100) | Acidobacteria_Gp7(100) | unclassified(100) | unclassified(100) | unclassified(100) |
| 147 | 350 | Proteobacteria(100) | Betaproteobacteria(100) | Burkholderiales(100) | Burkholderiales_inc. sedis(98) | unclassified(85) |
| 173 | 348 | unclassified(100) | unclassified(100) | unclassified(100) | unclassified(100) | unclassified(100) |
| 123 | 345 | Actinobacteria(100) | Actinobacteria(100) | Actinomycetales(100) | Geodermatophilaceae(100) | unclassified |
| 36 | 342 | Proteobacteria(100) | Alphaproteobacteria(88) | unclassified(88) | unclassified(88) | unclassified(88) |
| 96 | 337 | Nitrospira(100) | Nitrospira(100) | Nitrospirales(100) | Nitrospiraceae(100) | Nitrospira(100) |
| 254 | 332 | unclassified(100) | unclassified(100) | unclassified(100) | unclassified(100) | unclassified(100) |
| 309 | 317 | Actinobacteria(100) | Actinobacteria(100) | Actinomycetales(100) | Geodermatophilaceae(100) | Blastococcus(96) |
| 55 | 312 | Actinobacteria(100) | Actinobacteria(100) | Actinomycetales(100) | unclassified(100) | unclassified(100) |
| 191 | 312 | Actinobacteria(100) | Actinobacteria(100) | Solirubrobacterales(100) | unclassified(100) | unclassified(100) |
| 16 | 308 | Actinobacteria(100) | Actinobacteria(100) | Actinomycetales(100) | Streptomycetaceae(100) | Streptomyces(100) |
| 11 | 303 | unclassified(95) | unclassified(95) | unclassified(95) | unclassified(95) | unclassified(95) |
| 60 | 301 | Proteobacteria(100) | Alphaproteobacteria(100) | Rhodospirillales(100) | Acetobacteraceae(100) | unclassified(100) |
| 385 | 297 | Acidobacteria(100) | Acidobacteria_Gp4(100) | unclassified(100) | unclassified(100) | unclassified(100) |
| 7 | 291 | Actinobacteria(100) | Actinobacteria(100) | Actinomycetales(100) | Micrococcaceae(100) | Arthrobacter(100) |
| 43 | 287 | unclassified(100) | unclassified(100) | unclassified(100) | unclassified(100) | unclassified(100) |
| 8 | 280 | Firmicutes(100) | Bacilli(100) | Bacillales(100) | Bacillaceae(100) | Bacillus(100) |
| 14 | 278 | Actinobacteria(100) | Actinobacteria(100) | Actinomycetales(100) | unclassified(100) | unclassified(100) |
| 71 | 278 | Actinobacteria(100) | Actinobacteria(100) | Actinomycetales(100) | unclassified(100) | unclassified(100) |
| 137 | 276 | Proteobacteria(100) | Betaproteobacteria(100) | Burkholderiales(100) | Oxalobacteraceae(100) | unclassified |
| 1439 | 263 | unclassified(100) | unclassified(100) | unclassified(100) | unclassified(100) | unclassified(100) |
| 87 | 259 | Gemmatimonadetes(83) | Gemmatimonadetes(83) | Gemmatimonadales(83) | Gemmatimonadaceae(83) | Gemmatimonas(83) |
| 201 | 255 | Proteobacteria(100) | Alphaproteobacteria(88) | Rhizobiales(88) | unclassified | unclassified |
| 22 | 252 | Proteobacteria(100) | Alphaproteobacteria(100) | Rhizobiales(98) | unclassified(98) | unclassified(98) |
| 515 | 251 | unclassified(80) | unclassified(80) | unclassified(80) | unclassified(80) | unclassified(80) |
| 240 | 248 | Acidobacteria(100) | Acidobacteria_Gp16(100) | unclassified(100) | unclassified(100) | unclassified(100) |
| 69 | 241 | Proteobacteria(100) | Alphaproteobacteria(100) | Rhizobiales(100) | unclassified(99) | unclassified(99) |
| 169 | 240 | Proteobacteria(100) | unclassified(100) | unclassified(100) | unclassified(100) | unclassified(100) |
| 97 | 239 | Proteobacteria(100) | Alphaproteobacteria(100) | unclassified | unclassified | unclassified |
| 76 | 231 | Actinobacteria(100) | Actinobacteria(100) | Actinomycetales(100) | Streptomycetaceae(100) | unclassified |
| 32 | 230 | Actinobacteria(100) | Actinobacteria(100) | Actinomycetales(100) | Mycobacteriaceae(100) | Mycobacterium(100) |
| 273 | 228 | Actinobacteria(100) | Actinobacteria(100) | Actinomycetales(100) | Micromonosporaceae(100) | unclassified |
| 62 | 226 | Actinobacteria(100) | Actinobacteria(100) | Actinomycetales(100) | unclassified | unclassified |
| 355 | 225 | Bacteroidetes(100) | Sphingobacteria(100) | Sphingobacteriales(100) | Chitinophagaceae(100) | Terrimonas(86) |
| 1161 | 223 | Actinobacteria(100) | Actinobacteria(100) | Rubrobacterales(100) | Rubrobacteraceae(100) | Rubrobacter(100) |
| 39 | 222 | Proteobacteria(100) | Alphaproteobacteria(96) | unclassified(96) | unclassified(96) | unclassified(96) |
| 194 | 211 | Proteobacteria(100) | unclassified | unclassified | unclassified | unclassified |
| 192 | 210 | Acidobacteria(100) | Acidobacteria_Gp6(100) | unclassified(100) | unclassified(100) | unclassified(100) |
| 85 | 208 | Actinobacteria(100) | Actinobacteria(100) | Actinomycetales(100) | Pseudonocardiaceae(99) | Pseudonocardia(98) |
| 583 | 200 | Actinobacteria(100) | Actinobacteria(100) | Solirubrobacterales(100) | Solirubrobacteraceae(100) | Solirubrobacter(100) |
| 182 | 198 | Actinobacteria(100) | Actinobacteria(100) | Solirubrobacterales(100) | Solirubrobacteraceae(95) | Solirubrobacter(95) |
| 535 | 197 | Firmicutes(100) | Bacilli(100) | Bacillales(100) | Bacillaceae(100) | Bacillus(93) |
| 263 | 188 | Actinobacteria(100) | Actinobacteria(100) | Actinomycetales(100) | Propionibacteriaceae(100) | unclassified(97) |
| 88 | 187 | Proteobacteria(100) | Betaproteobacteria(100) | Burkholderiales(100) | Oxalobacteraceae(100) | unclassified |
| 86 | 183 | Proteobacteria(100) | Alphaproteobacteria(100) | Caulobacterales(91) | Caulobacteraceae(89) | unclassified |
| 113 | 180 | Acidobacteria(100) | Acidobacteria_Gp16(100) | unclassified(100) | unclassified(100) | unclassified(100) |
| 142 | 180 | Actinobacteria(100) | Actinobacteria(100) | Actinomycetales(100) | unclassified(100) | unclassified(100) |
| 356 | 178 | Actinobacteria(100) | Actinobacteria(100) | Actinomycetales(100) | Micromonosporaceae(100) | unclassified |
| 153 | 176 | Actinobacteria(100) | Actinobacteria(100) | Solirubrobacterales(100) | Solirubrobacteraceae(100) | Solirubrobacter(100) |
| 216 | 176 | unclassified(100) | unclassified(100) | unclassified(100) | unclassified(100) | unclassified(100) |
| 317 | 175 | Proteobacteria(100) | Alphaproteobacteria(100) | unclassified | unclassified | unclassified |
| 669 | 174 | unclassified(100) | unclassified(100) | unclassified(100) | unclassified(100) | unclassified(100) |
| 431 | 172 | Proteobacteria(100) | Gammaproteobacteria(99) | Xanthomonadales(94) | Xanthomonadaceae(94) | unclassified |
| 12 | 169 | Proteobacteria(100) | Alphaproteobacteria(100) | Rhizobiales(100) | unclassified(100) | unclassified(100) |
| 985 | 169 | Proteobacteria(100) | Alphaproteobacteria(100) | Caulobacterales(98) | Caulobacteraceae(98) | Caulobacter(98) |
| 380 | 162 | Actinobacteria(100) | Actinobacteria(100) | Solirubrobacterales(100) | unclassified(94) | unclassified(94) |
| 1417 | 162 | Actinobacteria(100) | Actinobacteria(100) | Actinomycetales(100) | Micromonosporaceae(100) | unclassified(100) |
| 257 | 158 | Acidobacteria(100) | Acidobacteria_Gp6(100) | unclassified(100) | unclassified(100) | unclassified(100) |
| 244 | 157 | unclassified | unclassified | unclassified | unclassified | unclassified |
| 100 | 156 | Proteobacteria(100) | Alphaproteobacteria(100) | Rhizobiales(100) | Methylobacteriaceae(92) | Methylobacterium(92) |
| 146 | 156 | unclassified(100) | unclassified(100) | unclassified(100) | unclassified(100) | unclassified(100) |
| 1520 | 154 | Actinobacteria(100) | Actinobacteria(100) | Solirubrobacterales(94) | unclassified(94) | unclassified(94) |
| 139 | 151 | Actinobacteria(100) | Actinobacteria(100) | Actinomycetales(100) | Mycobacteriaceae(97) | Mycobacterium(97) |
| 653 | 151 | Proteobacteria(100) | unclassified | unclassified | unclassified | unclassified |
| 1390 | 151 | Actinobacteria(100) | Actinobacteria(100) | Solirubrobacterales(100) | unclassified(100) | unclassified(100) |
| 82 | 150 | Proteobacteria(100) | unclassified | unclassified | unclassified | unclassified |
| 410 | 149 | Actinobacteria(100) | Actinobacteria(100) | Actinomycetales(100) | Nocardioidaceae(98) | Kribbella(98) |
| 597 | 147 | unclassified(96) | unclassified(96) | unclassified(96) | unclassified(96) | unclassified(96) |
| 23 | 144 | Proteobacteria(100) | Alphaproteobacteria(100) | Rhizobiales(100) | unclassified(100) | unclassified(100) |
| 2349 | 144 | Actinobacteria(100) | Actinobacteria(100) | Solirubrobacterales(95) | unclassified(95) | unclassified(95) |
| 378 | 142 | unclassified | unclassified | unclassified | unclassified | unclassified |
| 125 | 139 | Acidobacteria(100) | Acidobacteria_Gp4(100) | unclassified(100) | unclassified(100) | unclassified(100) |
| 95 | 138 | Proteobacteria(100) | Alphaproteobacteria(100) | Rhizobiales(100) | unclassified(96) | unclassified(96) |
| 49 | 136 | Bacteroidetes(100) | Sphingobacteria(100) | Sphingobacteriales(100) | Chitinophagaceae(100) | Flavisolibacter(86) |
| 47 | 133 | unclassified | unclassified | unclassified | unclassified | unclassified |
| 502 | 132 | Proteobacteria(100) | Alphaproteobacteria(100) | Rhizobiales(99) | unclassified(97) | unclassified(97) |
| 442 | 131 | Actinobacteria(100) | Actinobacteria(100) | Actinomycetales(100) | Micromonosporaceae(100) | unclassified(85) |
| 124 | 128 | Proteobacteria(100) | Alphaproteobacteria(100) | unclassified(100) | unclassified(100) | unclassified(100) |
| 519 | 127 | Proteobacteria(100) | Deltaproteobacteria(99) | Myxococcales(99) | unclassified(99) | unclassified(99) |
| 534 | 127 | Proteobacteria(100) | Alphaproteobacteria(100) | Rhizobiales(100) | unclassified | unclassified |
| 48 | 125 | Actinobacteria(100) | Actinobacteria(100) | Solirubrobacterales(100) | Patulibacteraceae(98) | Patulibacter(98) |
| 299 | 125 | Actinobacteria(100) | Actinobacteria(100) | Actinomycetales(100) | Nocardioidaceae(100) | unclassified |
| 151 | 124 | Actinobacteria(100) | Actinobacteria(100) | Actinomycetales(100) | Streptomycetaceae(100) | Streptomyces(100) |
| 166 | 122 | Proteobacteria(100) | Alphaproteobacteria(100) | Rhizobiales(100) | unclassified(100) | unclassified(100) |
| 101 | 120 | Actinobacteria(100) | Actinobacteria(100) | Actinomycetales(100) | Micromonosporaceae(100) | unclassified(91) |
| 308 | 120 | Proteobacteria(100) | Alphaproteobacteria(100) | Caulobacterales(100) | Caulobacteraceae(100) | Phenylobacterium(100) |
| 401 | 120 | Actinobacteria(100) | Actinobacteria(100) | Solirubrobacterales(100) | unclassified(100) | unclassified(100) |
| 679 | 120 | Proteobacteria(100) | Alphaproteobacteria(100) | Rhizobiales(100) | unclassified(100) | unclassified(100) |
| 428 | 119 | Acidobacteria(100) | Acidobacteria_Gp5(100) | unclassified(100) | unclassified(100) | unclassified(100) |
| 64 | 116 | Acidobacteria(100) | Acidobacteria_Gp1(100) | unclassified(100) | unclassified(100) | unclassified(100) |
| 154 | 115 | Proteobacteria(100) | Betaproteobacteria(100) | Burkholderiales(100) | Oxalobacteraceae(100) | Massilia(100) |
| 393 | 113 | Actinobacteria(100) | Actinobacteria(100) | Actinomycetales(100) | Thermomonosporaceae(100) | Actinoallomurus(100) |
| 206 | 111 | Proteobacteria(100) | Alphaproteobacteria(100) | Rhizobiales(91) | unclassified(91) | unclassified(91) |
| 457 | 111 | Proteobacteria(100) | Betaproteobacteria(100) | Burkholderiales(82) | unclassified(82) | unclassified(82) |
| 491 | 107 | Actinobacteria(100) | Actinobacteria(100) | Actinomycetales(100) | Nocardioidaceae(100) | Nocardioides(100) |
| 1176 | 107 | Acidobacteria(100) | Acidobacteria_Gp4(100) | unclassified(100) | unclassified(100) | unclassified(100) |
| 778 | 106 | Proteobacteria(100) | Betaproteobacteria(100) | unclassified(100) | unclassified(100) | unclassified(100) |
| 175 | 105 | Actinobacteria(100) | Actinobacteria(100) | Solirubrobacterales(100) | unclassified(86) | unclassified(86) |
| 285 | 105 | Acidobacteria(100) | Acidobacteria_Gp6(100) | unclassified(100) | unclassified(100) | unclassified(100) |
| 1475 | 105 | Proteobacteria(100) | Alphaproteobacteria(100) | Rhizobiales(100) | unclassified(99) | unclassified(99) |
| 61 | 104 | Proteobacteria(100) | Alphaproteobacteria(100) | Rhizobiales(100) | unclassified(100) | unclassified(100) |
| 1574 | 102 | Proteobacteria(100) | Alphaproteobacteria(100) | Rhizobiales(100) | unclassified(100) | unclassified(100) |
| 183 | 101 | Actinobacteria(100) | Actinobacteria(100) | Actinomycetales(100) | unclassified(100) | unclassified(100) |
| 104 | 99 | Actinobacteria(100) | Actinobacteria(100) | Solirubrobacterales(98) | unclassified(97) | unclassified(97) |
| 316 | 97 | Proteobacteria(100) | unclassified | unclassified | unclassified | unclassified |
| 1119 | 97 | Acidobacteria(100) | Acidobacteria_Gp16(100) | unclassified(100) | unclassified(100) | unclassified(100) |
| 221 | 96 | Actinobacteria(100) | Actinobacteria(100) | Solirubrobacterales(100) | unclassified(99) | unclassified(99) |
| 10 | 95 | Actinobacteria(100) | Actinobacteria(100) | Acidimicrobiales(90) | Iamiaceae(90) | Iamia(90) |
| 269 | 95 | Actinobacteria(100) | Actinobacteria(100) | Solirubrobacterales(100) | unclassified(99) | unclassified(99) |
| 231 | 93 | Acidobacteria(100) | Acidobacteria_Gp6(100) | unclassified(100) | unclassified(100) | unclassified(100) |
| 188 | 92 | unclassified | unclassified | unclassified | unclassified | unclassified |
| 209 | 91 | Nitrospira(100) | Nitrospira(100) | Nitrospirales(100) | Nitrospiraceae(100) | Nitrospira(100) |
| 90 | 90 | Proteobacteria(100) | Alphaproteobacteria(92) | unclassified(92) | unclassified(92) | unclassified(92) |
| 666 | 90 | unclassified(92) | unclassified(92) | unclassified(92) | unclassified(92) | unclassified(92) |
| 1461 | 88 | Acidobacteria(100) | Acidobacteria_Gp6(100) | unclassified(100) | unclassified(100) | unclassified(100) |
| 2246 | 88 | Actinobacteria(100) | Actinobacteria(100) | Actinomycetales(100) | Micromonosporaceae(100) | unclassified |
| 526 | 87 | Proteobacteria(100) | unclassified(100) | unclassified(100) | unclassified(100) | unclassified(100) |
| 2076 | 87 | unclassified(100) | unclassified(100) | unclassified(100) | unclassified(100) | unclassified(100) |
| 167 | 86 | Proteobacteria(100) | Alphaproteobacteria(91) | unclassified(91) | unclassified(91) | unclassified(91) |
| 255 | 85 | Actinobacteria(100) | Actinobacteria(100) | unclassified | unclassified | unclassified |
| 267 | 85 | Actinobacteria(100) | Actinobacteria(100) | Actinomycetales(100) | Nocardioidaceae(100) | Kribbella(100) |
| 157 | 84 | Proteobacteria(100) | Alphaproteobacteria(100) | Rhodospirillales(99) | Rhodospirillaceae(99) | Skermanella(99) |
| 421 | 82 | Actinobacteria(100) | Actinobacteria(100) | Solirubrobacterales(100) | unclassified(99) | unclassified(99) |
| 769 | 82 | Actinobacteria(100) | Actinobacteria(100) | Actinomycetales(100) | Nocardioidaceae(96) | Marmoricola(92) |
| 181 | 80 | Actinobacteria(100) | Actinobacteria(100) | Actinomycetales(100) | Propionibacteriaceae(100) | Microlunatus(100) |
| 537 | 80 | Actinobacteria(100) | Actinobacteria(100) | Solirubrobacterales(100) | Solirubrobacteraceae(100) | Solirubrobacter(100) |
| 333 | 78 | Acidobacteria(100) | Acidobacteria_Gp6(100) | unclassified(100) | unclassified(100) | unclassified(100) |
| 520 | 78 | Proteobacteria(100) | Deltaproteobacteria(100) | Myxococcales(99) | unclassified(88) | unclassified(88) |
| 615 | 78 | Actinobacteria(100) | Actinobacteria(100) | unclassified | unclassified | unclassified |
| 112 | 77 | Proteobacteria(100) | Alphaproteobacteria(100) | Rhodospirillales(100) | Rhodospirillaceae(100) | Skermanella(100) |
| 498 | 77 | Proteobacteria(100) | Gammaproteobacteria(100) | Xanthomonadales(100) | Xanthomonadaceae(100) | unclassified(100) |
| 782 | 76 | unclassified(82) | unclassified(82) | unclassified(82) | unclassified(82) | unclassified(82) |
| 177 | 75 | Actinobacteria(100) | Actinobacteria(100) | Actinomycetales(100) | Nocardioidaceae(95) | Marmoricola(84) |
| 107 | 74 | unclassified(100) | unclassified(100) | unclassified(100) | unclassified(100) | unclassified(100) |
| 507 | 74 | Proteobacteria(100) | Betaproteobacteria(99) | unclassified(99) | unclassified(99) | unclassified(99) |
| 618 | 74 | Planctomycetes(100) | Planctomycetacia(100) | Planctomycetales(100) | Planctomycetaceae(100) | Pirellula(99) |
| 866 | 74 | Proteobacteria(100) | unclassified | unclassified | unclassified | unclassified |
| 399 | 73 | Acidobacteria(100) | Acidobacteria_Gp16(100) | unclassified(100) | unclassified(100) | unclassified(100) |
| 187 | 72 | Actinobacteria(100) | Actinobacteria(100) | Solirubrobacterales(100) | Conexibacteraceae(100) | Conexibacter(100) |
| 236 | 71 | Actinobacteria(100) | Actinobacteria(100) | Actinomycetales(100) | Streptomycetaceae(96) | unclassified(86) |
| 150 | 70 | unclassified(90) | unclassified(90) | unclassified(90) | unclassified(90) | unclassified(90) |
| 1490 | 70 | unclassified | unclassified | unclassified | unclassified | unclassified |
| 1494 | 70 | Proteobacteria(100) | Alphaproteobacteria(100) | Rhizobiales(100) | unclassified(100) | unclassified(100) |
| 375 | 69 | Actinobacteria(82) | Actinobacteria(82) | unclassified(82) | unclassified(82) | unclassified(82) |
| 445 | 68 | Proteobacteria(100) | Alphaproteobacteria(100) | Sphingomonadales(100) | Erythrobacteraceae(99) | unclassified |
| 744 | 68 | Firmicutes(100) | Bacilli(100) | Bacillales(100) | Bacillaceae(100) | Bacillus(86) |
| 1603 | 68 | Gemmatimonadetes(95) | Gemmatimonadetes(95) | Gemmatimonadales(95) | Gemmatimonadaceae(95) | Gemmatimonas(95) |
| 426 | 67 | Proteobacteria(100) | Alphaproteobacteria(100) | Rhizobiales(98) | unclassified(98) | unclassified(98) |
| 197 | 66 | Proteobacteria(100) | Alphaproteobacteria(100) | unclassified | unclassified | unclassified |
| 1482 | 65 | Firmicutes(100) | Bacilli(100) | Bacillales(100) | Bacillaceae(100) | Bacillus(99) |
| 198 | 64 | Proteobacteria(100) | unclassified | unclassified | unclassified | unclassified |
| 796 | 64 | unclassified(100) | unclassified(100) | unclassified(100) | unclassified(100) | unclassified(100) |
| 516 | 63 | Actinobacteria(100) | Actinobacteria(100) | Actinomycetales(100) | Geodermatophilaceae(100) | unclassified(97) |
| 1325 | 63 | Proteobacteria(99) | unclassified(85) | unclassified(85) | unclassified(85) | unclassified(85) |
| 115 | 62 | Proteobacteria(100) | Betaproteobacteria(99) | unclassified | unclassified | unclassified |
| 178 | 62 | Proteobacteria(100) | Betaproteobacteria(96) | unclassified(96) | unclassified(96) | unclassified(96) |
| 383 | 61 | Acidobacteria(100) | Acidobacteria_Gp6(100) | unclassified(100) | unclassified(100) | unclassified(100) |
| 247 | 60 | unclassified(100) | unclassified(100) | unclassified(100) | unclassified(100) | unclassified(100) |
| 453 | 60 | Actinobacteria(100) | Actinobacteria(100) | Actinomycetales(100) | Nocardioidaceae(94) | unclassified |
| 965 | 59 | Acidobacteria(97) | Acidobacteria_Gp7(97) | unclassified(97) | unclassified(97) | unclassified(97) |
| 1723 | 59 | Proteobacteria(100) | Alphaproteobacteria(100) | Rhodospirillales(100) | Acetobacteraceae(100) | Roseomonas(100) |
| 172 | 58 | unclassified(100) | unclassified(100) | unclassified(100) | unclassified(100) | unclassified(100) |
| 541 | 57 | Actinobacteria(100) | Actinobacteria(100) | unclassified(100) | unclassified(100) | unclassified(100) |
| 779 | 57 | Actinobacteria(100) | Actinobacteria(100) | Actinomycetales(100) | Micromonosporaceae(100) | unclassified(81) |
| 740 | 56 | unclassified(100) | unclassified(100) | unclassified(100) | unclassified(100) | unclassified(100) |
| 754 | 56 | Acidobacteria(100) | Acidobacteria_Gp6(100) | unclassified(100) | unclassified(100) | unclassified(100) |
| 671 | 55 | Bacteroidetes(100) | Sphingobacteria(100) | Sphingobacteriales(100) | Chitinophagaceae(100) | unclassified |
| 719 | 55 | Actinobacteria(100) | Actinobacteria(100) | Solirubrobacterales(100) | unclassified(100) | unclassified(100) |
| 1329 | 55 | Actinobacteria(100) | Actinobacteria(100) | Actinomycetales(100) | Nocardioidaceae(99) | Nocardioides(99) |
| 675 | 54 | Actinobacteria(100) | Actinobacteria(100) | Solirubrobacterales(100) | unclassified(100) | unclassified(100) |
| 2487 | 54 | Proteobacteria(99) | Gammaproteobacteria(93) | unclassified(91) | unclassified(91) | unclassified(91) |
| 614 | 52 | unclassified(100) | unclassified(100) | unclassified(100) | unclassified(100) | unclassified(100) |
| 999 | 51 | unclassified(100) | unclassified(100) | unclassified(100) | unclassified(100) | unclassified(100) |
| 1425 | 51 | unclassified(99) | unclassified(99) | unclassified(99) | unclassified(99) | unclassified(99) |
| 758 | 50 | Actinobacteria(98) | Actinobacteria(98) | unclassified | unclassified | unclassified |
| 841 | 50 | Gemmatimonadetes(96) | Gemmatimonadetes(96) | Gemmatimonadales(96) | Gemmatimonadaceae(96) | Gemmatimonas(96) |
| 844 | 50 | Acidobacteria(100) | Acidobacteria_Gp4(100) | unclassified(100) | unclassified(100) | unclassified(100) |
| 1517 | 50 | unclassified(100) | unclassified(100) | unclassified(100) | unclassified(100) | unclassified(100) |
| 1357 | 49 | Proteobacteria(100) | Alphaproteobacteria(100) | unclassified | unclassified | unclassified |
| 2761 | 49 | Actinobacteria(100) | Actinobacteria(100) | Actinomycetales(100) | unclassified(100) | unclassified(100) |
| 219 | 48 | Actinobacteria(100) | Actinobacteria(100) | Actinomycetales(100) | Micromonosporaceae(100) | unclassified(100) |
| 425 | 48 | Proteobacteria(100) | Betaproteobacteria(84) | unclassified(84) | unclassified(84) | unclassified(84) |
| 174 | 47 | Proteobacteria(100) | Betaproteobacteria(98) | unclassified(98) | unclassified(98) | unclassified(98) |
| 723 | 47 | Proteobacteria(100) | Deltaproteobacteria(86) | Myxococcales(86) | unclassified(86) | unclassified(86) |
| 1722 | 47 | Actinobacteria(100) | Actinobacteria(100) | unclassified | unclassified | unclassified |
| 386 | 46 | Actinobacteria(100) | Actinobacteria(100) | Actinomycetales(100) | Streptosporangiaceae(94) | unclassified |
| 1196 | 44 | Bacteroidetes(100) | Sphingobacteria(100) | Sphingobacteriales(100) | Chitinophagaceae(100) | unclassified(100) |
| 1518 | 41 | Proteobacteria(100) | Betaproteobacteria(100) | Nitrosomonadales(100) | Nitrosomonadaceae(100) | Nitrosospira(98) |
| 1590 | 41 | Proteobacteria(100) | Alphaproteobacteria(100) | Rhizobiales(100) | unclassified | unclassified |
| 415 | 39 | Proteobacteria(100) | Alphaproteobacteria(100) | Rhizobiales(100) | unclassified(100) | unclassified(100) |
| 808 | 39 | Proteobacteria(100) | Alphaproteobacteria(95) | unclassified(95) | unclassified(95) | unclassified(95) |
| 1492 | 39 | Actinobacteria(100) | Actinobacteria(100) | Solirubrobacterales(100) | unclassified(100) | unclassified(100) |
| 67 | 38 | Firmicutes(100) | Bacilli(100) | Bacillales(100) | Bacillaceae(100) | Bacillus(100) |
| 227 | 38 | unclassified(100) | unclassified(100) | unclassified(100) | unclassified(100) | unclassified(100) |
| 205 | 37 | Actinobacteria(100) | Actinobacteria(100) | Solirubrobacterales(100) | Solirubrobacteraceae(98) | Solirubrobacter(98) |
| 371 | 36 | Actinobacteria(100) | Actinobacteria(100) | Actinomycetales(100) | Micromonosporaceae(100) | unclassified |
| 468 | 36 | Actinobacteria(100) | Actinobacteria(100) | Solirubrobacterales(100) | unclassified(100) | unclassified(100) |
| 1379 | 36 | Proteobacteria(100) | Betaproteobacteria(100) | Burkholderiales(100) | Alcaligenaceae(100) | Azohydromonas(100) |
| 1530 | 36 | Proteobacteria(100) | Betaproteobacteria(98) | unclassified(98) | unclassified(98) | unclassified(98) |
| 681 | 35 | Actinobacteria(100) | Actinobacteria(100) | Actinomycetales(100) | Nocardioidaceae(100) | Nocardioides(100) |
| 1550 | 34 | Gemmatimonadetes(100) | Gemmatimonadetes(100) | Gemmatimonadales(100) | Gemmatimonadaceae(100) | Gemmatimonas(100) |
| 1581 | 34 | unclassified(100) | unclassified(100) | unclassified(100) | unclassified(100) | unclassified(100) |
| 243 | 33 | Proteobacteria(100) | Alphaproteobacteria(100) | Caulobacterales(100) | Caulobacteraceae(100) | unclassified |
| 880 | 33 | Bacteroidetes(100) | Sphingobacteria(100) | Sphingobacteriales(100) | Chitinophagaceae(100) | unclassified(100) |
| 1145 | 33 | Gemmatimonadetes(100) | Gemmatimonadetes(100) | Gemmatimonadales(100) | Gemmatimonadaceae(100) | Gemmatimonas(100) |
| 129 | 32 | Proteobacteria(100) | Alphaproteobacteria(100) | Rhizobiales(100) | Methylobacteriaceae(97) | Methylobacterium(97) |
| 774 | 32 | Acidobacteria(100) | Acidobacteria_Gp11(100) | unclassified(100) | unclassified(100) | unclassified(100) |
| 1780 | 32 | Actinobacteria(100) | Actinobacteria(100) | Actinomycetales(100) | unclassified(100) | unclassified(100) |
| 602 | 30 | Proteobacteria(100) | Alphaproteobacteria(94) | unclassified(94) | unclassified(94) | unclassified(94) |
| 264 | 29 | Actinobacteria(100) | Actinobacteria(100) | Solirubrobacterales(100) | unclassified | unclassified |
| 1424 | 28 | Actinobacteria(100) | Actinobacteria(100) | unclassified | unclassified | unclassified |
| 1682 | 26 | Actinobacteria(100) | Actinobacteria(100) | Actinomycetales(100) | Cryptosporangiaceae(85) | Cryptosporangium(85) |
| 1831 | 26 | Proteobacteria(100) | Alphaproteobacteria(100) | Rhodospirillales(100) | Acetobacteraceae(100) | unclassified(100) |
| 1192 | 25 | Acidobacteria(100) | Acidobacteria_Gp1(100) | unclassified(100) | unclassified(100) | unclassified(100) |
| 1582 | 25 | unclassified(100) | unclassified(100) | unclassified(100) | unclassified(100) | unclassified(100) |
| 2069 | 25 | Proteobacteria(96) | unclassified(96) | unclassified(96) | unclassified(96) | unclassified(96) |
| 2737 | 23 | Actinobacteria(100) | Actinobacteria(100) | Actinomycetales(100) | Micromonosporaceae(100) | Micromonospora(96) |
| 2879 | 23 | Acidobacteria(100) | Acidobacteria_Gp6(100) | unclassified(100) | unclassified(100) | unclassified(100) |
| 469 | 22 | Actinobacteria(100) | Actinobacteria(100) | Solirubrobacterales(100) | unclassified(100) | unclassified(100) |
| 660 | 21 | Firmicutes(100) | Bacilli(100) | Bacillales(100) | Bacillaceae(100) | Bacillus(100) |
| 767 | 20 | Actinobacteria(100) | Actinobacteria(100) | Actinomycetales(100) | unclassified(100) | unclassified(100) |
| 1601 | 18 | Actinobacteria(100) | Actinobacteria(100) | Solirubrobacterales(100) | unclassified(100) | unclassified(100) |
