# Supplementary material for: Bacterial Indicator of Agricultural Management for Soil under No-Till Crop Production
Source: PLoS One. 2012 Nov 30;7(11):e51075. doi: 10.1371/journal.pone.0051075 (PMC3511350; doi:10.1371/journal.pone.0051075)
Supplement: Table S4 — List of OTUs only common to good no-till agricultural practices (GAP) in the four locations. Sequences were assigned to taxonomic groups using the RDP classifier (http://rdp.cme.msu.edu/classifier/classifier.jsp). OTUs were sorted by the total number of sequences in the complete data set. (DOCX) [file pone.0051075.s007.docx]

Table S4: List of OTUs only common to good no-till agricultural practices (GAP) in the four locations. Sequences were assigned to taxonomic groups using the RDP classiﬁer (<http://rdp.cme.msu.edu/classifier/classifier.jsp>). OTUs were sorted by the total number of sequences in the complete data set.

| **OTU ID** | **Size** | **Phylum** | **Class** | **Order** | **Family** | **Genus** |
| --- | --- | --- | --- | --- | --- | --- |
| 5 | 338 | Firmicutes(100) | Bacilli(100) | Bacillales(100) | Bacillaceae(100) | unclassified(100) |
| 186 | 211 | Actinobacteria(100) | Actinobacteria(100) | Actinomycetales(100) | unclassified(100) | unclassified(100) |
| 1689 | 138 | Proteobacteria(100) | Gammaproteobacteria(100) | Xanthomonadales(100) | Xanthomonadaceae(100) | unclassified(100) |
| 742 | 135 | Actinobacteria(100) | Actinobacteria(100) | Actinomycetales(100) | Micromonosporaceae(99) | unclassified(84) |
| 1742 | 135 | unclassified(100) | unclassified(100) | unclassified(100) | unclassified(100) | unclassified(100) |
| 152 | 123 | Actinobacteria(100) | Actinobacteria(100) | unclassified | unclassified | unclassified |
| 446 | 119 | Acidobacteria(100) | Acidobacteria_Gp1(100) | unclassified(100) | unclassified(100) | unclassified(100) |
| 13 | 115 | Proteobacteria(100) | Alphaproteobacteria(100) | Rhizobiales(100) | unclassified(99) | unclassified(99) |
| 1335 | 110 | Actinobacteria(100) | Actinobacteria(100) | Actinomycetales(100) | Propionibacteriaceae(100) | Microlunatus(93) |
| 46 | 109 | Proteobacteria(100) | Betaproteobacteria(100) | Burkholderiales(100) | Burkholderiaceae(100) | Burkholderia(100) |
| 764 | 107 | Proteobacteria(100) | Deltaproteobacteria(98) | unclassified(98) | unclassified(98) | unclassified(98) |
| 916 | 103 | Actinobacteria(100) | Actinobacteria(100) | Solirubrobacterales(100) | unclassified(100) | unclassified(100) |
| 73 | 100 | Acidobacteria(100) | Acidobacteria_Gp1(100) | unclassified(100) | unclassified(100) | unclassified(100) |
| 397 | 100 | Bacteroidetes(100) | Sphingobacteria(100) | Sphingobacteriales(100) | Chitinophagaceae(100) | Terrimonas(100) |
| 283 | 94 | Proteobacteria(100) | Gammaproteobacteria(100) | Xanthomonadales(100) | Xanthomonadaceae(100) | unclassified(100) |
| 74 | 82 | Proteobacteria(100) | Alphaproteobacteria(100) | Rhizobiales(100) | Hyphomicrobiaceae(96) | unclassified(96) |
| 1967 | 80 | Actinobacteria(100) | Actinobacteria(100) | Solirubrobacterales(100) | Solirubrobacteraceae(100) | Solirubrobacter(100) |
| 546 | 72 | Acidobacteria(100) | Acidobacteria_Gp4(100) | unclassified(100) | unclassified(100) | unclassified(100) |
| 1411 | 72 | Acidobacteria(100) | Acidobacteria_Gp6(100) | unclassified(100) | unclassified(100) | unclassified(100) |
| 357 | 71 | Acidobacteria(100) | Acidobacteria_Gp6(100) | unclassified(100) | unclassified(100) | unclassified(100) |
| 708 | 71 | Actinobacteria(100) | Actinobacteria(100) | Actinomycetales(100) | Micromonosporaceae(100) | unclassified(100) |
| 239 | 68 | Actinobacteria(100) | Actinobacteria(100) | Actinomycetales(100) | Pseudonocardiaceae(100) | Amycolatopsis(99) |
| 633 | 68 | Actinobacteria(100) | Actinobacteria(100) | Actinomycetales(100) | Micromonosporaceae(100) | unclassified(100) |
| 3051 | 66 | Bacteroidetes(100) | Sphingobacteria(100) | Sphingobacteriales(100) | Chitinophagaceae(100) | Terrimonas(100) |
| 343 | 64 | Actinobacteria(100) | Actinobacteria(100) | Actinomycetales(100) | unclassified(100) | unclassified(100) |
| 810 | 63 | Actinobacteria(100) | Actinobacteria(100) | Actinomycetales(100) | unclassified(100) | unclassified(100) |
| 523 | 62 | Proteobacteria(100) | Betaproteobacteria(100) | Burkholderiales(100) | unclassified(100) | unclassified(100) |
| 934 | 61 | unclassified(100) | unclassified(100) | unclassified(100) | unclassified(100) | unclassified(100) |
| 1431 | 61 | Actinobacteria(100) | Actinobacteria(100) | Actinomycetales(100) | Pseudonocardiaceae(89) | unclassified |
| 1605 | 61 | Actinobacteria(100) | Actinobacteria(100) | Actinomycetales(100) | Nocardioidaceae(100) | Nocardioides(99) |
| 297 | 59 | Firmicutes(100) | Bacilli(100) | Bacillales(100) | Paenibacillaceae(100) | Paenibacillus(100) |
| 559 | 58 | Actinobacteria(100) | Actinobacteria(100) | Actinomycetales(100) | Pseudonocardiaceae(100) | Actinomycetospora(90) |
| 644 | 56 | Bacteroidetes(100) | Sphingobacteria(100) | Sphingobacteriales(100) | Chitinophagaceae(100) | unclassified |
| 3 | 55 | Proteobacteria(100) | Alphaproteobacteria(100) | unclassified | unclassified | unclassified |
| 695 | 55 | Acidobacteria(100) | Acidobacteria_Gp6(100) | unclassified(100) | unclassified(100) | unclassified(100) |
| 1436 | 54 | Actinobacteria(100) | Actinobacteria(100) | Actinomycetales(100) | Thermomonosporaceae(100) | Actinoallomurus(100) |
| 89 | 50 | Actinobacteria(100) | Actinobacteria(100) | Actinomycetales(100) | Mycobacteriaceae(96) | Mycobacterium(96) |
| 512 | 50 | Actinobacteria(100) | Actinobacteria(100) | Actinomycetales(100) | Micromonosporaceae(100) | unclassified |
| 1419 | 50 | Proteobacteria(100) | unclassified | unclassified | unclassified | unclassified |
| 1725 | 50 | Proteobacteria(100) | Alphaproteobacteria(100) | Rhodospirillales(100) | Acetobacteraceae(100) | unclassified(90) |
| 643 | 49 | unclassified(100) | unclassified(100) | unclassified(100) | unclassified(100) | unclassified(100) |
| 1636 | 49 | Acidobacteria(100) | Acidobacteria_Gp1(100) | unclassified(100) | unclassified(100) | unclassified(100) |
| 351 | 48 | Proteobacteria(100) | Alphaproteobacteria(100) | unclassified(100) | unclassified(100) | unclassified(100) |
| 694 | 48 | Acidobacteria(100) | Acidobacteria_Gp1(100) | unclassified(100) | unclassified(100) | unclassified(100) |
| 846 | 48 | Acidobacteria(100) | Acidobacteria_Gp6(100) | unclassified(100) | unclassified(100) | unclassified(100) |
| 1454 | 47 | Acidobacteria(100) | Acidobacteria_Gp1(100) | unclassified(100) | unclassified(100) | unclassified(100) |
| 180 | 45 | Actinobacteria(100) | Actinobacteria(100) | Actinomycetales(100) | Micromonosporaceae(100) | unclassified |
| 563 | 45 | Acidobacteria(100) | Acidobacteria_Gp2(100) | unclassified(100) | unclassified(100) | unclassified(100) |
| 585 | 45 | Proteobacteria(100) | Alphaproteobacteria(100) | Rhizobiales(100) | Hyphomicrobiaceae(100) | Devosia(87) |
| 994 | 44 | Proteobacteria(100) | Alphaproteobacteria(100) | unclassified(100) | unclassified(100) | unclassified(100) |
| 161 | 43 | Actinobacteria(100) | Actinobacteria(100) | Actinomycetales(100) | Micromonosporaceae(100) | unclassified(100) |
| 655 | 42 | Actinobacteria(100) | Actinobacteria(100) | Actinomycetales(100) | unclassified(100) | unclassified(100) |
| 613 | 41 | Bacteroidetes(100) | Sphingobacteria(100) | Sphingobacteriales(100) | Chitinophagaceae(100) | unclassified |
| 159 | 39 | Bacteroidetes(100) | Sphingobacteria(100) | Sphingobacteriales(100) | Chitinophagaceae(100) | unclassified(98) |
| 800 | 39 | Acidobacteria(100) | Acidobacteria_Gp1(100) | unclassified(100) | unclassified(100) | unclassified(100) |
| 2996 | 39 | Actinobacteria(100) | Actinobacteria(100) | Actinomycetales(100) | unclassified(100) | unclassified(100) |
| 2920 | 38 | Actinobacteria(100) | Actinobacteria(100) | Actinomycetales(100) | Nocardiaceae(100) | Rhodococcus(100) |
| 684 | 37 | Proteobacteria(90) | unclassified | unclassified | unclassified | unclassified |
| 582 | 36 | Proteobacteria(100) | Alphaproteobacteria(100) | Rhizobiales(100) | Methylobacteriaceae(100) | Methylobacterium(100) |
| 623 | 36 | Actinobacteria(100) | Actinobacteria(100) | Actinomycetales(100) | Micromonosporaceae(100) | unclassified(100) |
| 886 | 35 | Proteobacteria(100) | Alphaproteobacteria(100) | Rhizobiales(100) | Bradyrhizobiaceae(100) | Bosea(100) |
| 1608 | 35 | Actinobacteria(100) | Actinobacteria(100) | Actinomycetales(100) | Microbacteriaceae(100) | Agromyces(100) |
| 2397 | 35 | Gemmatimonadetes(92) | Gemmatimonadetes(92) | Gemmatimonadales(92) | Gemmatimonadaceae(92) | Gemmatimonas(92) |
| 449 | 34 | Actinobacteria(100) | Actinobacteria(100) | Acidimicrobiales(100) | Iamiaceae(100) | Iamia(100) |
| 721 | 34 | Proteobacteria(100) | Alphaproteobacteria(100) | Sphingomonadales(100) | Sphingomonadaceae(100) | unclassified |
| 987 | 34 | Acidobacteria(100) | Acidobacteria_Gp1(100) | unclassified(100) | unclassified(100) | unclassified(100) |
| 2986 | 34 | Acidobacteria(100) | Acidobacteria_Gp6(100) | unclassified(100) | unclassified(100) | unclassified(100) |
| 84 | 33 | Proteobacteria(100) | Alphaproteobacteria(100) | Rhodospirillales(100) | Acetobacteraceae(100) | unclassified(100) |
| 836 | 33 | Proteobacteria(100) | Alphaproteobacteria(100) | Sphingomonadales(100) | Sphingomonadaceae(100) | unclassified |
| 975 | 33 | Actinobacteria(100) | Actinobacteria(100) | Actinomycetales(100) | Microbacteriaceae(100) | unclassified |
| 1247 | 33 | Proteobacteria(100) | Alphaproteobacteria(100) | Rhizobiales(100) | Methylobacteriaceae(100) | Methylobacterium(100) |
| 1557 | 33 | Actinobacteria(100) | Actinobacteria(100) | Actinomycetales(100) | unclassified(100) | unclassified(100) |
| 2044 | 33 | Actinobacteria(100) | Actinobacteria(100) | Solirubrobacterales(100) | unclassified | unclassified |
| 608 | 32 | Acidobacteria(100) | Acidobacteria_Gp16(100) | unclassified(100) | unclassified(100) | unclassified(100) |
| 1011 | 30 | unclassified(97) | unclassified(97) | unclassified(97) | unclassified(97) | unclassified(97) |
| 411 | 29 | Acidobacteria(100) | Acidobacteria_Gp6(100) | unclassified(100) | unclassified(100) | unclassified(100) |
| 894 | 29 | Actinobacteria(100) | Actinobacteria(100) | Actinomycetales(100) | Nocardioidaceae(94) | Aeromicrobium(94) |
| 165 | 28 | Planctomycetes(100) | Planctomycetacia(100) | Planctomycetales(100) | Planctomycetaceae(100) | unclassified(100) |
| 256 | 28 | Acidobacteria(100) | Acidobacteria_Gp3(100) | unclassified(100) | unclassified(100) | unclassified(100) |
| 286 | 28 | Acidobacteria(100) | Acidobacteria_Gp6(100) | unclassified(100) | unclassified(100) | unclassified(100) |
| 805 | 28 | unclassified | unclassified | unclassified | unclassified | unclassified |
| 403 | 27 | Proteobacteria(100) | Alphaproteobacteria(100) | Rhizobiales(100) | unclassified(100) | unclassified(100) |
| 2937 | 27 | Actinobacteria(100) | Actinobacteria(100) | Actinomycetales(100) | unclassified(100) | unclassified(100) |
| 500 | 26 | Bacteroidetes(100) | Sphingobacteria(100) | Sphingobacteriales(100) | Chitinophagaceae(100) | unclassified |
| 924 | 26 | unclassified | unclassified | unclassified | unclassified | unclassified |
| 1264 | 26 | Actinobacteria(100) | Actinobacteria(100) | Actinomycetales(100) | Streptomycetaceae(100) | Streptomyces(100) |
| 1446 | 26 | unclassified(100) | unclassified(100) | unclassified(100) | unclassified(100) | unclassified(100) |
| 1502 | 26 | Bacteroidetes(100) | Sphingobacteria(100) | Sphingobacteriales(100) | Chitinophagaceae(100) | unclassified(100) |
| 91 | 25 | Actinobacteria(100) | Actinobacteria(100) | Actinomycetales(100) | Microbacteriaceae(100) | Microbacterium(100) |
| 326 | 25 | Actinobacteria(100) | Actinobacteria(100) | Solirubrobacterales(100) | unclassified(88) | unclassified(88) |
| 528 | 25 | Acidobacteria(100) | Acidobacteria_Gp2(100) | unclassified(100) | unclassified(100) | unclassified(100) |
| 1421 | 25 | Acidobacteria(100) | Acidobacteria_Gp1(100) | unclassified(100) | unclassified(100) | unclassified(100) |
| 143 | 24 | Actinobacteria(100) | Actinobacteria(100) | Actinomycetales(100) | Micrococcaceae(100) | Arthrobacter(100) |
| 2002 | 24 | Bacteroidetes(100) | Sphingobacteria(100) | Sphingobacteriales(100) | Chitinophagaceae(100) | unclassified(100) |
| 3133 | 24 | Proteobacteria(100) | Alphaproteobacteria(100) | Rhodospirillales(96) | Rhodospirillaceae(96) | Skermanella(96) |
| 565 | 23 | Planctomycetes(100) | Planctomycetacia(100) | Planctomycetales(100) | Planctomycetaceae(100) | Zavarzinella(100) |
| 819 | 23 | Actinobacteria(100) | Actinobacteria(100) | Solirubrobacterales(92) | unclassified(92) | unclassified(92) |
| 1509 | 23 | unclassified(100) | unclassified(100) | unclassified(100) | unclassified(100) | unclassified(100) |
| 287 | 21 | Gemmatimonadetes(100) | Gemmatimonadetes(100) | Gemmatimonadales(100) | Gemmatimonadaceae(100) | Gemmatimonas(100) |
| 529 | 21 | unclassified(100) | unclassified(100) | unclassified(100) | unclassified(100) | unclassified(100) |
| 762 | 21 | Proteobacteria(100) | Alphaproteobacteria(100) | Rhizobiales(100) | unclassified(81) | unclassified(81) |
| 862 | 21 | Actinobacteria(100) | Actinobacteria(100) | Actinomycetales(100) | Micromonosporaceae(100) | unclassified(100) |
| 2005 | 21 | Actinobacteria(100) | Actinobacteria(100) | Acidimicrobiales(100) | Iamiaceae(100) | Iamia(100) |
| 222 | 20 | Proteobacteria(100) | Betaproteobacteria(100) | Burkholderiales(100) | Burkholderiaceae(100) | Burkholderia(100) |
| 363 | 19 | Planctomycetes(100) | Planctomycetacia(100) | Planctomycetales(100) | Planctomycetaceae(100) | Zavarzinella(100) |
| 373 | 19 | Acidobacteria(100) | Acidobacteria_Gp6(100) | unclassified(100) | unclassified(100) | unclassified(100) |
| 761 | 19 | Proteobacteria(100) | Betaproteobacteria(95) | Burkholderiales(90) | unclassified(90) | unclassified(90) |
| 1304 | 19 | unclassified | unclassified | unclassified | unclassified | unclassified |
| 1541 | 19 | unclassified(100) | unclassified(100) | unclassified(100) | unclassified(100) | unclassified(100) |
| 1584 | 19 | Proteobacteria(100) | Alphaproteobacteria(100) | Rhizobiales(100) | unclassified(100) | unclassified(100) |
| 1733 | 19 | Proteobacteria(100) | unclassified(100) | unclassified(100) | unclassified(100) | unclassified(100) |
| 2999 | 19 | Acidobacteria(100) | Acidobacteria_Gp5(100) | unclassified(100) | unclassified(100) | unclassified(100) |
| 266 | 18 | Bacteroidetes(100) | Sphingobacteria(100) | Sphingobacteriales(100) | Chitinophagaceae(100) | Flavisolibacter(100) |
| 636 | 18 | Actinobacteria(100) | Actinobacteria(100) | unclassified | unclassified | unclassified |
| 1190 | 18 | Actinobacteria(100) | Actinobacteria(100) | Actinomycetales(100) | Micromonosporaceae(89) | unclassified(89) |
| 128 | 17 | Proteobacteria(100) | Alphaproteobacteria(100) | Rhizobiales(100) | unclassified(100) | unclassified(100) |
| 1686 | 17 | Proteobacteria(100) | Gammaproteobacteria(100) | Xanthomonadales(100) | Xanthomonadaceae(100) | unclassified(100) |
| 2398 | 17 | Proteobacteria(100) | Alphaproteobacteria(100) | Rhodospirillales(100) | Rhodospirillaceae(100) | Inquilinus(100) |
| 2887 | 17 | Bacteroidetes(100) | Sphingobacteria(100) | Sphingobacteriales(100) | unclassified(100) | unclassified(100) |
| 1727 | 16 | Actinobacteria(100) | Actinobacteria(100) | Actinomycetales(100) | Propionibacteriaceae(100) | unclassified(100) |
| 1207 | 15 | Proteobacteria(100) | Alphaproteobacteria(100) | Rhizobiales(100) | unclassified(87) | unclassified(87) |
| 1503 | 15 | Acidobacteria(100) | Acidobacteria_Gp16(100) | unclassified(100) | unclassified(100) | unclassified(100) |
| 1765 | 15 | Acidobacteria(100) | Acidobacteria_Gp5(100) | unclassified(100) | unclassified(100) | unclassified(100) |
| 1993 | 15 | unclassified(100) | unclassified(100) | unclassified(100) | unclassified(100) | unclassified(100) |
| 2103 | 15 | Actinobacteria(100) | Actinobacteria(100) | Rubrobacterales(100) | Rubrobacteraceae(100) | Rubrobacter(100) |
| 2624 | 15 | Proteobacteria(100) | Deltaproteobacteria(100) | Myxococcales(100) | Polyangiaceae(87) | unclassified |
| 2733 | 15 | unclassified(100) | unclassified(100) | unclassified(100) | unclassified(100) | unclassified(100) |
| 3099 | 15 | Proteobacteria(100) | Alphaproteobacteria(100) | Rhizobiales(100) | Beijerinckiaceae(100) | unclassified(100) |
| 703 | 14 | Actinobacteria(100) | Actinobacteria(100) | Actinomycetales(100) | Pseudonocardiaceae(100) | Pseudonocardia(86) |
| 1254 | 14 | Actinobacteria(100) | Actinobacteria(100) | Solirubrobacterales(100) | Solirubrobacteraceae(100) | Solirubrobacter(100) |
| 1678 | 14 | Actinobacteria(100) | Actinobacteria(100) | Actinomycetales(100) | unclassified(100) | unclassified(100) |
| 852 | 13 | Actinobacteria(100) | Actinobacteria(100) | Actinomycetales(100) | Micromonosporaceae(100) | unclassified(100) |
| 973 | 13 | Bacteroidetes(100) | Sphingobacteria(100) | Sphingobacteriales(100) | Chitinophagaceae(100) | unclassified(100) |
| 1773 | 13 | Acidobacteria(100) | Acidobacteria_Gp6(100) | unclassified(100) | unclassified(100) | unclassified(100) |
| 2822 | 13 | Acidobacteria(100) | Acidobacteria_Gp6(100) | unclassified(100) | unclassified(100) | unclassified(100) |
| 575 | 12 | Planctomycetes(100) | Planctomycetacia(100) | Planctomycetales(100) | Planctomycetaceae(100) | unclassified(100) |
| 1265 | 12 | Planctomycetes(100) | Planctomycetacia(100) | Planctomycetales(100) | Planctomycetaceae(100) | Pirellula(100) |
| 2505 | 12 | Proteobacteria(100) | Alphaproteobacteria(100) | Rhodospirillales(100) | Acetobacteraceae(100) | unclassified(100) |
| 2687 | 12 | Actinobacteria(100) | Actinobacteria(100) | Solirubrobacterales(100) | unclassified(92) | unclassified(92) |
| 612 | 11 | Firmicutes(100) | Bacilli(100) | Bacillales(100) | unclassified | unclassified |
| 1123 | 10 | Gemmatimonadetes(100) | Gemmatimonadetes(100) | Gemmatimonadales(100) | Gemmatimonadaceae(100) | Gemmatimonas(100) |
| 3109 | 9 | Firmicutes(100) | Bacilli(100) | Bacillales(100) | Paenibacillaceae(100) | Paenibacillus(100) |
| 2809 | 7 | Proteobacteria(100) | Gammaproteobacteria(100) | Xanthomonadales(100) | Xanthomonadaceae(100) | unclassified(100) |
