# Supplementary material for: Bacterial Indicator of Agricultural Management for Soil under No-Till Crop Production
Source: PLoS One. 2012 Nov 30;7(11):e51075. doi: 10.1371/journal.pone.0051075 (PMC3511350; doi:10.1371/journal.pone.0051075)
Supplement: Table S5 — List of OTUs common only to poor no-till agricultural practices (PAP) in the four locations. Sequences were assigned to taxonomic groups using the RDP classifier (http://rdp.cme.msu.edu/classifier/classifier.jsp). OTUs were sorted by the total number of sequences in the complete data set. (DOCX) [file pone.0051075.s008.docx]

Table S5: List of OTUs common only to poor no-till agricultural practices (PAP) in the four locations. Sequences were assigned to taxonomic groups using the RDP classiﬁer (<http://rdp.cme.msu.edu/classifier/classifier.jsp>). OTUs were sorted by the total number of sequences in the complete data set.

| **OTUID** | **Size** | **Phylum** | **Class** | **Order** | **Family** | **Genus** |
| --- | --- | --- | --- | --- | --- | --- |
| 3146 | 235 | Actinobacteria(100) | Actinobacteria(100) | Actinomycetales(100) | Propionibacteriaceae(100) | unclassified(99) |
| 3310 | 215 | unclassified(100) | unclassified(100) | unclassified(100) | unclassified(100) | unclassified(100) |
| 3252 | 206 | Verrucomicrobia(100) | Spartobacteria(100) | unclassified(100) | unclassified(100) | unclassified(100) |
| 59 | 187 | Proteobacteria(100) | Alphaproteobacteria(98) | unclassified(98) | unclassified(98) | unclassified(98) |
| 65 | 176 | Firmicutes(100) | Bacilli(100) | Bacillales(100) | Bacillaceae(100) | unclassified |
| 45 | 135 | Proteobacteria(100) | Betaproteobacteria(100) | Burkholderiales(100) | Burkholderiaceae(100) | Burkholderia(100) |
| 51 | 135 | Actinobacteria(100) | Actinobacteria(100) | Actinomycetales(100) | unclassified(100) | unclassified(100) |
| 44 | 125 | Actinobacteria(100) | Actinobacteria(100) | unclassified(99) | unclassified(99) | unclassified(99) |
| 4207 | 113 | Proteobacteria(100) | Alphaproteobacteria(100) | Rhizobiales(100) | Hyphomicrobiaceae(93) | Rhodoplanes(93) |
| 53 | 101 | Proteobacteria(100) | Alphaproteobacteria(100) | Rhizobiales(100) | Hyphomicrobiaceae(99) | unclassified |
| 40 | 96 | Actinobacteria(100) | Actinobacteria(100) | Actinomycetales(100) | Pseudonocardiaceae(92) | unclassified(92) |
| 3585 | 96 | Actinobacteria(100) | Actinobacteria(100) | Actinomycetales(100) | Intrasporangiaceae(100) | unclassified(100) |
| 459 | 92 | Acidobacteria(100) | Acidobacteria_Gp6(100) | unclassified(100) | unclassified(100) | unclassified(100) |
| 3601 | 86 | Proteobacteria(100) | Betaproteobacteria(100) | Burkholderiales(100) | Comamonadaceae(100) | Variovorax(98) |
| 1572 | 84 | Actinobacteria(100) | Actinobacteria(100) | Solirubrobacterales(100) | unclassified | unclassified |
| 315 | 83 | Actinobacteria(100) | Actinobacteria(100) | Actinomycetales(100) | Actinosynnemataceae(100) | unclassified |
| 361 | 77 | unclassified(90) | unclassified(90) | unclassified(90) | unclassified(90) | unclassified(90) |
| 92 | 76 | Proteobacteria(100) | Alphaproteobacteria(100) | Rhizobiales(100) | unclassified(97) | unclassified(97) |
| 368 | 76 | Proteobacteria(100) | Alphaproteobacteria(100) | Rhizobiales(100) | unclassified(100) | unclassified(100) |
| 578 | 76 | unclassified(81) | unclassified(81) | unclassified(81) | unclassified(81) | unclassified(81) |
| 1498 | 76 | Proteobacteria(100) | Alphaproteobacteria(100) | Rhodobacterales(100) | Rhodobacteraceae(100) | Rubellimicrobium(100) |
| 452 | 74 | Actinobacteria(100) | Actinobacteria(100) | Solirubrobacterales(100) | unclassified(100) | unclassified(100) |
| 338 | 72 | unclassified(100) | unclassified(100) | unclassified(100) | unclassified(100) | unclassified(100) |
| 170 | 71 | Proteobacteria(100) | Alphaproteobacteria(99) | unclassified(99) | unclassified(99) | unclassified(99) |
| 1290 | 66 | Actinobacteria(100) | Actinobacteria(100) | Actinomycetales(100) | Micromonosporaceae(100) | unclassified(81) |
| 3162 | 66 | Bacteroidetes(100) | Sphingobacteria(100) | Sphingobacteriales(100) | Chitinophagaceae(100) | unclassified(100) |
| 261 | 65 | Acidobacteria(100) | Acidobacteria_Gp6(100) | unclassified(100) | unclassified(100) | unclassified(100) |
| 340 | 65 | Proteobacteria(100) | Betaproteobacteria(100) | unclassified(100) | unclassified(100) | unclassified(100) |
| 460 | 64 | Acidobacteria(100) | Acidobacteria_Gp16(100) | unclassified(100) | unclassified(100) | unclassified(100) |
| 148 | 62 | unclassified(100) | unclassified(100) | unclassified(100) | unclassified(100) | unclassified(100) |
| 450 | 61 | Actinobacteria(100) | Actinobacteria(100) | Actinomycetales(100) | Micromonosporaceae(100) | unclassified(100) |
| 215 | 60 | unclassified(100) | unclassified(100) | unclassified(100) | unclassified(100) | unclassified(100) |
| 3594 | 59 | Actinobacteria(100) | Actinobacteria(100) | Actinomycetales(100) | Mycobacteriaceae(100) | Mycobacterium(100) |
| 303 | 57 | Actinobacteria(100) | Actinobacteria(100) | Actinomycetales(100) | unclassified(100) | unclassified(100) |
| 503 | 57 | Actinobacteria(100) | Actinobacteria(100) | Actinomycetales(100) | Nocardioidaceae(100) | Nocardioides(97) |
| 1522 | 57 | unclassified | unclassified | unclassified | unclassified | unclassified |
| 3584 | 57 | Actinobacteria(100) | Actinobacteria(100) | Solirubrobacterales(86) | unclassified(81) | unclassified(81) |
| 80 | 55 | Actinobacteria(100) | Actinobacteria(100) | Actinomycetales(100) | unclassified(100) | unclassified(100) |
| 93 | 55 | Actinobacteria(100) | Actinobacteria(100) | Solirubrobacterales(100) | unclassified(100) | unclassified(100) |
| 203 | 55 | unclassified(100) | unclassified(100) | unclassified(100) | unclassified(100) | unclassified(100) |
| 350 | 55 | Actinobacteria(100) | Actinobacteria(100) | Actinomycetales(100) | Intrasporangiaceae(100) | Terrabacter(97) |
| 741 | 55 | Actinobacteria(100) | Actinobacteria(100) | Actinomycetales(100) | Streptomycetaceae(100) | Streptomyces(100) |
| 138 | 54 | Actinobacteria(100) | Actinobacteria(100) | Actinomycetales(100) | unclassified(99) | unclassified(99) |
| 207 | 53 | Proteobacteria(100) | Alphaproteobacteria(100) | Rhizobiales(100) | Phyllobacteriaceae(100) | unclassified |
| 1405 | 53 | Acidobacteria(100) | Acidobacteria_Gp5(100) | unclassified(100) | unclassified(100) | unclassified(100) |
| 875 | 52 | Proteobacteria(100) | Alphaproteobacteria(100) | Rhizobiales(100) | unclassified(100) | unclassified(100) |
| 1087 | 52 | Proteobacteria(100) | Alphaproteobacteria(100) | Rhizobiales(99) | unclassified(89) | unclassified(89) |
| 857 | 50 | Gemmatimonadetes(98) | Gemmatimonadetes(98) | Gemmatimonadales(98) | Gemmatimonadaceae(98) | Gemmatimonas(98) |
| 1527 | 50 | Proteobacteria(100) | Alphaproteobacteria(100) | Rhizobiales(100) | unclassified(100) | unclassified(100) |
| 5226 | 49 | unclassified(82) | unclassified(82) | unclassified(82) | unclassified(82) | unclassified(82) |
| 463 | 48 | Gemmatimonadetes(100) | Gemmatimonadetes(100) | Gemmatimonadales(100) | Gemmatimonadaceae(100) | Gemmatimonas(100) |
| 34 | 47 | Proteobacteria(100) | Alphaproteobacteria(100) | Rhizobiales(100) | unclassified | unclassified |
| 799 | 46 | unclassified | unclassified | unclassified | unclassified | unclassified |
| 252 | 45 | Acidobacteria(100) | Acidobacteria_Gp7(100) | unclassified(100) | unclassified(100) | unclassified(100) |
| 668 | 43 | Acidobacteria(100) | Acidobacteria_Gp7(100) | unclassified(100) | unclassified(100) | unclassified(100) |
| 438 | 42 | Proteobacteria(100) | Alphaproteobacteria(100) | Rhizobiales(100) | Methylobacteriaceae(81) | Microvirga(81) |
| 490 | 42 | Acidobacteria(100) | Acidobacteria_Gp4(100) | unclassified(100) | unclassified(100) | unclassified(100) |
| 265 | 41 | Proteobacteria(100) | Betaproteobacteria(100) | unclassified | unclassified | unclassified |
| 509 | 41 | Actinobacteria(100) | Actinobacteria(100) | unclassified | unclassified | unclassified |
| 888 | 41 | Actinobacteria(100) | Actinobacteria(100) | Actinomycetales(100) | Nocardioidaceae(100) | Nocardioides(98) |
| 1516 | 41 | Proteobacteria(100) | Alphaproteobacteria(100) | Rhizobiales(100) | unclassified(100) | unclassified(100) |
| 2935 | 41 | Actinobacteria(100) | Actinobacteria(100) | Actinomycetales(100) | unclassified(100) | unclassified(100) |
| 525 | 40 | Actinobacteria(100) | Actinobacteria(100) | Solirubrobacterales(100) | Conexibacteraceae(83) | Conexibacter(83) |
| 4208 | 40 | Proteobacteria(100) | Deltaproteobacteria(100) | unclassified | unclassified | unclassified |
| 482 | 39 | Acidobacteria(100) | Acidobacteria_Gp6(100) | unclassified(100) | unclassified(100) | unclassified(100) |
| 1487 | 39 | Actinobacteria(100) | Actinobacteria(100) | unclassified | unclassified | unclassified |
| 3285 | 39 | unclassified(100) | unclassified(100) | unclassified(100) | unclassified(100) | unclassified(100) |
| 2817 | 38 | Actinobacteria(100) | Actinobacteria(100) | Actinomycetales(100) | Streptomycetaceae(100) | Streptomyces(95) |
| 656 | 37 | Bacteroidetes(100) | Sphingobacteria(100) | Sphingobacteriales(100) | Chitinophagaceae(100) | unclassified(100) |
| 1354 | 37 | Proteobacteria(100) | Alphaproteobacteria(100) | Sphingomonadales(100) | Sphingomonadaceae(100) | unclassified |
| 217 | 36 | Proteobacteria(100) | Betaproteobacteria(100) | Burkholderiales(100) | Burkholderiaceae(100) | Cupriavidus(100) |
| 432 | 36 | unclassified(98) | unclassified(98) | unclassified(98) | unclassified(98) | unclassified(98) |
| 731 | 36 | Actinobacteria(100) | Actinobacteria(100) | Actinomycetales(100) | Micromonosporaceae(100) | unclassified(100) |
| 1285 | 36 | unclassified(98) | unclassified(98) | unclassified(98) | unclassified(98) | unclassified(98) |
| 1470 | 36 | Actinobacteria(100) | Actinobacteria(100) | Solirubrobacterales(100) | unclassified(100) | unclassified(100) |
| 524 | 35 | Proteobacteria(100) | Alphaproteobacteria(100) | Rhizobiales(100) | unclassified(100) | unclassified(100) |
| 554 | 35 | unclassified(89) | unclassified(89) | unclassified(89) | unclassified(89) | unclassified(89) |
| 753 | 35 | Nitrospira(100) | Nitrospira(100) | Nitrospirales(100) | Nitrospiraceae(100) | Nitrospira(100) |
| 1599 | 35 | Actinobacteria(100) | Actinobacteria(100) | Solirubrobacterales(100) | unclassified | unclassified |
| 324 | 34 | Proteobacteria(100) | Alphaproteobacteria(100) | Rhizobiales(100) | unclassified(100) | unclassified(100) |
| 416 | 34 | Actinobacteria(100) | Actinobacteria(100) | Solirubrobacterales(100) | unclassified(100) | unclassified(100) |
| 1156 | 34 | Actinobacteria(100) | Actinobacteria(100) | Actinomycetales(100) | Micromonosporaceae(100) | unclassified(100) |
| 3666 | 34 | Proteobacteria(100) | Deltaproteobacteria(100) | unclassified | unclassified | unclassified |
| 108 | 33 | Actinobacteria(100) | Actinobacteria(100) | Actinomycetales(100) | Streptomycetaceae(100) | Streptomyces(100) |
| 195 | 33 | Proteobacteria(100) | Betaproteobacteria(100) | Burkholderiales(100) | Oxalobacteraceae(100) | Herbaspirillum(85) |
| 1013 | 33 | Proteobacteria(100) | unclassified | unclassified | unclassified | unclassified |
| 2522 | 33 | Proteobacteria(100) | Alphaproteobacteria(100) | Rhizobiales(100) | unclassified | unclassified |
| 2715 | 33 | unclassified(100) | unclassified(100) | unclassified(100) | unclassified(100) | unclassified(100) |
| 3227 | 33 | Proteobacteria(100) | unclassified | unclassified | unclassified | unclassified |
| 3906 | 33 | Acidobacteria(100) | Acidobacteria_Gp6(100) | unclassified(100) | unclassified(100) | unclassified(100) |
| 881 | 32 | Acidobacteria(100) | Acidobacteria_Gp16(100) | unclassified(100) | unclassified(100) | unclassified(100) |
| 1402 | 32 | Acidobacteria(100) | Acidobacteria_Gp6(100) | unclassified(100) | unclassified(100) | unclassified(100) |
| 3597 | 32 | unclassified(100) | unclassified(100) | unclassified(100) | unclassified(100) | unclassified(100) |
| 1552 | 31 | Verrucomicrobia(100) | Spartobacteria(100) | unclassified(100) | unclassified(100) | unclassified(100) |
| 2578 | 31 | Actinobacteria(100) | Actinobacteria(100) | Actinomycetales(100) | unclassified | unclassified |
| 2781 | 31 | Actinobacteria(100) | Actinobacteria(100) | Actinomycetales(100) | Streptomycetaceae(100) | Streptomyces(97) |
| 877 | 30 | Acidobacteria(100) | Acidobacteria_Gp4(100) | unclassified(100) | unclassified(100) | unclassified(100) |
| 1092 | 30 | Acidobacteria(100) | Acidobacteria_Gp4(100) | unclassified(100) | unclassified(100) | unclassified(100) |
| 1903 | 30 | Acidobacteria(100) | Acidobacteria_Gp6(100) | unclassified(100) | unclassified(100) | unclassified(100) |
| 2417 | 30 | Proteobacteria(100) | Alphaproteobacteria(100) | Rhizobiales(100) | unclassified | unclassified |
| 2632 | 30 | unclassified(100) | unclassified(100) | unclassified(100) | unclassified(100) | unclassified(100) |
| 111 | 29 | Proteobacteria(100) | Deltaproteobacteria(100) | unclassified(100) | unclassified(100) | unclassified(100) |
| 3112 | 29 | Bacteroidetes(100) | Sphingobacteria(100) | Sphingobacteriales(100) | Chitinophagaceae(100) | unclassified(100) |
| 5230 | 29 | Actinobacteria(100) | Actinobacteria(100) | unclassified(97) | unclassified(97) | unclassified(97) |
| 171 | 28 | Actinobacteria(100) | Actinobacteria(100) | Actinomycetales(100) | Nocardioidaceae(100) | unclassified(86) |
| 752 | 28 | unclassified(100) | unclassified(100) | unclassified(100) | unclassified(100) | unclassified(100) |
| 1340 | 28 | Proteobacteria(100) | Alphaproteobacteria(100) | Sphingomonadales(97) | Sphingomonadaceae(83) | unclassified(83) |
| 1513 | 28 | Acidobacteria(100) | Acidobacteria_Gp6(100) | unclassified(100) | unclassified(100) | unclassified(100) |
| 1551 | 28 | Actinobacteria(100) | Actinobacteria(100) | Actinomycetales(100) | unclassified(100) | unclassified(100) |
| 1764 | 28 | Acidobacteria(100) | Acidobacteria_Gp16(100) | unclassified(100) | unclassified(100) | unclassified(100) |
| 2065 | 28 | unclassified(93) | unclassified(93) | unclassified(93) | unclassified(93) | unclassified(93) |
| 5705 | 28 | Acidobacteria(100) | Acidobacteria_Gp6(100) | unclassified(100) | unclassified(100) | unclassified(100) |
| 600 | 27 | Acidobacteria(89) | Acidobacteria_Gp7(89) | unclassified(89) | unclassified(89) | unclassified(89) |
| 1268 | 27 | Proteobacteria(100) | Alphaproteobacteria(100) | Rhodospirillales(100) | Acetobacteraceae(100) | unclassified(100) |
| 2484 | 27 | Actinobacteria(100) | Actinobacteria(100) | Actinomycetales(100) | Micromonosporaceae(100) | unclassified(100) |
| 3129 | 27 | Actinobacteria(100) | Actinobacteria(100) | Solirubrobacterales(100) | Solirubrobacteraceae(100) | Solirubrobacter(100) |
| 202 | 26 | unclassified(100) | unclassified(100) | unclassified(100) | unclassified(100) | unclassified(100) |
| 2226 | 26 | Proteobacteria(100) | Deltaproteobacteria(100) | Myxococcales(97) | unclassified(97) | unclassified(97) |
| 3355 | 26 | unclassified(100) | unclassified(100) | unclassified(100) | unclassified(100) | unclassified(100) |
| 4580 | 26 | Proteobacteria(100) | Alphaproteobacteria(97) | unclassified(97) | unclassified(97) | unclassified(97) |
| 160 | 25 | unclassified(100) | unclassified(100) | unclassified(100) | unclassified(100) | unclassified(100) |
| 659 | 25 | Actinobacteria(100) | Actinobacteria(100) | Actinomycetales(100) | Micromonosporaceae(100) | unclassified(100) |
| 3274 | 25 | Actinobacteria(100) | Actinobacteria(100) | Actinomycetales(100) | Pseudonocardiaceae(100) | Pseudonocardia(100) |
| 558 | 24 | Acidobacteria(84) | Acidobacteria_Gp7(84) | unclassified(84) | unclassified(84) | unclassified(84) |
| 974 | 24 | unclassified | unclassified | unclassified | unclassified | unclassified |
| 4423 | 24 | Proteobacteria(100) | Alphaproteobacteria(100) | Rhizobiales(100) | unclassified(100) | unclassified(100) |
| 4551 | 24 | Acidobacteria(100) | Acidobacteria_Gp4(100) | unclassified(100) | unclassified(100) | unclassified(100) |
| 4834 | 24 | Actinobacteria(100) | Actinobacteria(100) | Rubrobacterales(100) | Rubrobacteraceae(100) | Rubrobacter(100) |
| 185 | 23 | Actinobacteria(100) | Actinobacteria(100) | Actinomycetales(100) | Intrasporangiaceae(100) | unclassified(87) |
| 424 | 23 | Actinobacteria(100) | Actinobacteria(100) | Actinomycetales(100) | unclassified(100) | unclassified(100) |
| 513 | 23 | Actinobacteria(100) | Actinobacteria(100) | unclassified(100) | unclassified(100) | unclassified(100) |
| 1047 | 23 | Actinobacteria(100) | Actinobacteria(100) | Solirubrobacterales(100) | unclassified(87) | unclassified(87) |
| 1111 | 23 | Proteobacteria(100) | Betaproteobacteria(100) | unclassified | unclassified | unclassified |
| 1348 | 23 | Actinobacteria(100) | Actinobacteria(100) | Actinomycetales(100) | Micromonosporaceae(100) | unclassified |
| 1726 | 23 | Nitrospira(100) | Nitrospira(100) | Nitrospirales(100) | Nitrospiraceae(100) | Nitrospira(100) |
| 3555 | 23 | Proteobacteria(100) | Alphaproteobacteria(100) | Sphingomonadales(100) | Sphingomonadaceae(100) | unclassified |
| 4431 | 23 | Proteobacteria(100) | unclassified(100) | unclassified(100) | unclassified(100) | unclassified(100) |
| 1172 | 22 | Proteobacteria(100) | Alphaproteobacteria(100) | Sphingomonadales(82) | unclassified | unclassified |
| 1750 | 22 | Actinobacteria(100) | Actinobacteria(100) | Solirubrobacterales(100) | unclassified(100) | unclassified(100) |
| 3668 | 22 | Actinobacteria(100) | Actinobacteria(100) | Actinomycetales(100) | unclassified(100) | unclassified(100) |
| 435 | 21 | Acidobacteria(100) | Acidobacteria_Gp6(100) | unclassified(100) | unclassified(100) | unclassified(100) |
| 1274 | 21 | unclassified | unclassified | unclassified | unclassified | unclassified |
| 2000 | 21 | Acidobacteria(100) | Acidobacteria_Gp4(100) | unclassified(100) | unclassified(100) | unclassified(100) |
| 3921 | 21 | Actinobacteria(100) | Actinobacteria(100) | Actinomycetales(100) | Nocardiaceae(91) | Nocardia(86) |
| 294 | 20 | Proteobacteria(100) | unclassified(90) | unclassified(90) | unclassified(90) | unclassified(90) |
| 492 | 20 | Actinobacteria(100) | Actinobacteria(100) | Actinomycetales(100) | Micromonosporaceae(100) | unclassified(100) |
| 942 | 20 | unclassified | unclassified | unclassified | unclassified | unclassified |
| 1149 | 20 | Acidobacteria(95) | Acidobacteria_Gp7(90) | unclassified(90) | unclassified(90) | unclassified(90) |
| 1561 | 20 | unclassified | unclassified | unclassified | unclassified | unclassified |
| 1734 | 20 | Actinobacteria(100) | Actinobacteria(100) | Actinomycetales(100) | Micromonosporaceae(100) | unclassified(85) |
| 3490 | 20 | Actinobacteria(100) | Actinobacteria(100) | Actinomycetales(100) | unclassified(100) | unclassified(100) |
| 4983 | 20 | Actinobacteria(100) | Actinobacteria(100) | unclassified(100) | unclassified(100) | unclassified(100) |
| 8186 | 20 | unclassified(100) | unclassified(100) | unclassified(100) | unclassified(100) | unclassified(100) |
| 929 | 19 | Bacteroidetes(100) | Sphingobacteria(100) | Sphingobacteriales(100) | Chitinophagaceae(100) | Terrimonas(90) |
| 1060 | 19 | TM7(100) | unclassified(100) | unclassified(100) | unclassified(100) | unclassified(100) |
| 1445 | 19 | unclassified(100) | unclassified(100) | unclassified(100) | unclassified(100) | unclassified(100) |
| 1980 | 19 | Acidobacteria(95) | Acidobacteria_Gp7(95) | unclassified(95) | unclassified(95) | unclassified(95) |
| 2543 | 19 | Proteobacteria(100) | Deltaproteobacteria(100) | Myxococcales(100) | Polyangiaceae(100) | unclassified |
| 478 | 18 | unclassified(100) | unclassified(100) | unclassified(100) | unclassified(100) | unclassified(100) |
| 3581 | 18 | unclassified | unclassified | unclassified | unclassified | unclassified |
| 4313 | 18 | unclassified(95) | unclassified(95) | unclassified(95) | unclassified(95) | unclassified(95) |
| 4960 | 18 | Acidobacteria(100) | Acidobacteria_Gp4(100) | unclassified(100) | unclassified(100) | unclassified(100) |
| 5994 | 18 | Actinobacteria(100) | Actinobacteria(100) | Solirubrobacterales(100) | unclassified(95) | unclassified(95) |
| 711 | 17 | Actinobacteria(100) | Actinobacteria(100) | unclassified | unclassified | unclassified |
| 1856 | 17 | Actinobacteria(100) | Actinobacteria(100) | Solirubrobacterales(100) | Solirubrobacteraceae(100) | Solirubrobacter(100) |
| 3369 | 17 | Actinobacteria(100) | Actinobacteria(100) | Solirubrobacterales(100) | unclassified(100) | unclassified(100) |
| 3402 | 17 | unclassified(100) | unclassified(100) | unclassified(100) | unclassified(100) | unclassified(100) |
| 4366 | 17 | Verrucomicrobia(100) | Spartobacteria(100) | unclassified(100) | unclassified(100) | unclassified(100) |
| 4764 | 17 | Proteobacteria(100) | Betaproteobacteria(100) | Burkholderiales(100) | Oxalobacteraceae(89) | unclassified |
| 342 | 16 | Proteobacteria(100) | Alphaproteobacteria(100) | Rhizobiales(100) | Methylobacteriaceae(100) | Methylobacterium(100) |
| 603 | 16 | unclassified(100) | unclassified(100) | unclassified(100) | unclassified(100) | unclassified(100) |
| 811 | 16 | Acidobacteria(100) | Acidobacteria_Gp4(100) | unclassified(100) | unclassified(100) | unclassified(100) |
| 1747 | 16 | unclassified(94) | unclassified(94) | unclassified(94) | unclassified(94) | unclassified(94) |
| 2259 | 16 | Verrucomicrobia(100) | Spartobacteria(100) | unclassified(100) | unclassified(100) | unclassified(100) |
| 1084 | 15 | Actinobacteria(100) | Actinobacteria(100) | Actinomycetales(100) | Actinosynnemataceae(80) | unclassified |
| 1144 | 15 | unclassified(100) | unclassified(100) | unclassified(100) | unclassified(100) | unclassified(100) |
| 5833 | 15 | unclassified(100) | unclassified(100) | unclassified(100) | unclassified(100) | unclassified(100) |
| 1493 | 14 | unclassified(100) | unclassified(100) | unclassified(100) | unclassified(100) | unclassified(100) |
| 4842 | 14 | Actinobacteria(100) | Actinobacteria(100) | Solirubrobacterales(100) | unclassified(100) | unclassified(100) |
| 392 | 13 | unclassified(100) | unclassified(100) | unclassified(100) | unclassified(100) | unclassified(100) |
| 1690 | 13 | TM7(100) | unclassified(100) | unclassified(100) | unclassified(100) | unclassified(100) |
| 1946 | 13 | Proteobacteria(100) | Betaproteobacteria(100) | unclassified(100) | unclassified(100) | unclassified(100) |
| 2008 | 13 | Bacteroidetes(100) | Sphingobacteria(100) | Sphingobacteriales(100) | Chitinophagaceae(100) | unclassified(93) |
| 665 | 12 | Proteobacteria(100) | Betaproteobacteria(92) | unclassified(92) | unclassified(92) | unclassified(92) |
| 865 | 12 | Bacteroidetes(100) | Sphingobacteria(100) | Sphingobacteriales(100) | Chitinophagaceae(100) | Flavisolibacter(84) |
| 1775 | 12 | unclassified(100) | unclassified(100) | unclassified(100) | unclassified(100) | unclassified(100) |
| 1898 | 12 | Bacteroidetes(100) | Sphingobacteria(100) | Sphingobacteriales(100) | Chitinophagaceae(100) | unclassified(100) |
| 347 | 11 | unclassified(100) | unclassified(100) | unclassified(100) | unclassified(100) | unclassified(100) |
| 818 | 11 | unclassified(100) | unclassified(100) | unclassified(100) | unclassified(100) | unclassified(100) |
| 3947 | 11 | Proteobacteria(100) | Deltaproteobacteria(100) | Myxococcales(100) | Cystobacteraceae(100) | unclassified(91) |
| 4319 | 11 | unclassified | unclassified | unclassified | unclassified | unclassified |
| 4560 | 11 | Actinobacteria(100) | Actinobacteria(100) | unclassified(100) | unclassified(100) | unclassified(100) |
| 6649 | 11 | Actinobacteria(100) | Actinobacteria(100) | Actinomycetales(100) | Micromonosporaceae(91) | unclassified(91) |
| 7397 | 10 | Actinobacteria(100) | Actinobacteria(100) | Solirubrobacterales(100) | Solirubrobacteraceae(100) | Solirubrobacter(100) |
| 7766 | 10 | Proteobacteria(100) | Alphaproteobacteria(100) | Rhodospirillales(100) | Rhodospirillaceae(100) | Skermanella(100) |
| 1179 | 9 | unclassified(100) | unclassified(100) | unclassified(100) | unclassified(100) | unclassified(100) |
| 1952 | 7 | Acidobacteria(100) | Acidobacteria_Gp4(100) | unclassified(100) | unclassified(100) | unclassified(100) |
| 3763 | 7 | Acidobacteria(100) | Acidobacteria_Gp6(100) | unclassified(100) | unclassified(100) | unclassified(100) |
| 8180 | 7 | unclassified(100) | unclassified(100) | unclassified(100) | unclassified(100) | unclassified(100) |
| 4620 | 5 | Actinobacteria(100) | Actinobacteria(100) | Actinomycetales(100) | Propionibacteriaceae(100) | Microlunatus(80) |
| 8836 | 5 | unclassified(100) | unclassified(100) | unclassified(100) | unclassified(100) | unclassified(100) |
