# Supplementary material for: Bacterial Indicator of Agricultural Management for Soil under No-Till Crop Production
Source: PLoS One. 2012 Nov 30;7(11):e51075. doi: 10.1371/journal.pone.0051075 (PMC3511350; doi:10.1371/journal.pone.0051075)
Supplement: Table S6 — Results of indicator species analysis. For each of the taxa, we indicate the Indicator Value index (IndVal), the number of samples that contain the taxon (Freq), the statistical significance of the association (p-value), the total number of sequences corresponding to the OTU (size), and the lowest taxonomic rank assigned with a bootstrap confidence indicated in parenthesis. Agricultural managements GAP and PAP an NE are defined in the main text. Only OTUs with IndVal higher than 0.75 are shown. (DOCX) [file pone.0051075.s009.docx]

Table S6: Results of indicator species analysis. For each of the taxa, we indicate the Indicator Value index (IndVal), the number of samples that contain the taxon (Freq), the statistical signiﬁcance of the association (p-value), the total number of sequences corresponding to the OTU (size), and the lowest taxonomic rank assigned with a bootstrap confidence indicated in parenthesis. Agricultural managements GAP and PAP an NE are deﬁned in the main text. Only OTUs with IndVal higher than 0.75 are shown.

| **Group** | **OUT ID** | **IndVal** | **p** | **Freq** | **Size** | **Phylum** | **Class** | **Order** | **Family** | **Genus** |
| --- | --- | --- | --- | --- | --- | --- | --- | --- | --- | --- |
| GAP | 3109 | 1.000 | 0.008 | 4 | 9 | Firmicutes(100) | Bacilli(100) | Bacillales(100) | Paenibacillaceae(100) | Paenibacillus(100) |
| GAP | 3 | 0.909 | 0.035 | 7 | 55 | Proteobacteria(100) | Alphaproteobacteria(100) | unclassified | unclassified | unclassified |
| GAP | 73 | 0.860 | 0.022 | 8 | 100 | Acidobacteria(100) | Acidobacteria_Gp1(100) | unclassified(100) | unclassified(100) | unclassified(100) |
| GAP | 2809 | 0.857 | 0.022 | 5 | 7 | Proteobacteria(100) | Gammaproteobacteria(100) | Xanthomonadales(100) | Xanthomonadaceae(100) | unclassified(100) |
| GAP | 1446 | 0.846 | 0.02 | 7 | 26 | unclassified(100) | unclassified(100) | unclassified(100) | unclassified(100) | unclassified(100) |
| GAP | 373 | 0.842 | 0.012 | 6 | 19 | Acidobacteria(100) | Acidobacteria_Gp6(100) | unclassified(100) | unclassified(100) | unclassified(100) |
| GAP | 1509 | 0.826 | 0.034 | 6 | 23 | unclassified(100) | unclassified(100) | unclassified(100) | unclassified(100) | unclassified(100) |
| GAP | 612 | 0.818 | 0.04 | 6 | 11 | Firmicutes(100) | Bacilli(100) | Bacillales(100) | unclassified | unclassified |
| GAP | 1123 | 0.800 | 0.023 | 6 | 10 | Gemmatimonadetes(100) | Gemmatimonadetes(100) | Gemmatimonadales(100) | Gemmatimonadaceae(100) | Gemmatimonas(100) |
| GAP | 2733 | 0.800 | 0.07 | 7 | 15 | unclassified(100) | unclassified(100) | unclassified(100) | unclassified(100) | unclassified(100) |
| GAP | 608 | 0.781 | 0.073 | 8 | 32 | Acidobacteria(100) | Acidobacteria_Gp16(100) | unclassified(100) | unclassified(100) | unclassified(100) |
| GAP | 128 | 0.765 | 0.058 | 7 | 17 | Proteobacteria(100) | Alphaproteobacteria(100) | Rhizobiales(100) | unclassified(100) | unclassified(100) |
| GAP | 1224 | 0.750 | 0.046 | 3 | 5 | Bacteroidetes(100) | Sphingobacteria(100) | Sphingobacteriales(100) | Chitinophagaceae(100) | unclassified(100) |
| GAP | 2205 | 0.750 | 0.046 | 3 | 5 | Proteobacteria(100) | unclassified(100) | unclassified(100) | unclassified(100) | unclassified(100) |
| GAP | 2613 | 0.750 | 0.047 | 3 | 5 | unclassified(100) | unclassified(100) | unclassified(100) | unclassified(100) | unclassified(100) |
| GAP | 1345 | 0.750 | 0.048 | 3 | 3 | Chloroflexi(100) | Thermomicrobia(100) | Sphaerobacterales(100) | Sphaerobacteraceae(100) | Sphaerobacter(100) |
| GAP | 3455 | 0.750 | 0.05 | 3 | 4 | unclassified(100) | unclassified(100) | unclassified(100) | unclassified(100) | unclassified(100) |
| GAP | 1139 | 0.750 | 0.052 | 3 | 4 | Proteobacteria(100) | Alphaproteobacteria(100) | Rhodospirillales(100) | Acetobacteraceae(100) | unclassified(100) |
| GAP | 2871 | 0.750 | 0.052 | 3 | 3 | Proteobacteria(100) | Deltaproteobacteria(100) | Myxococcales(100) | unclassified(100) | unclassified(100) |
| GAP | 3721 | 0.750 | 0.054 | 3 | 4 | Bacteroidetes(100) | unclassified(100) | unclassified(100) | unclassified(100) | unclassified(100) |
| GAP | 3753 | 0.750 | 0.055 | 3 | 3 | Bacteroidetes(100) | Flavobacteria(100) | Flavobacteriales(100) | Flavobacteriaceae(100) | unclassified |
| GAP | 1762 | 0.750 | 0.059 | 3 | 9 | unclassified(100) | unclassified(100) | unclassified(100) | unclassified(100) | unclassified(100) |
| GAP | 2410 | 0.750 | 0.061 | 3 | 6 | Actinobacteria(100) | Actinobacteria(100) | unclassified(100) | unclassified(100) | unclassified(100) |
| GAP | 4127 | 0.750 | 0.062 | 3 | 3 | Actinobacteria(100) | Actinobacteria(100) | Acidimicrobiales(100) | Iamiaceae(100) | Iamia(100) |
| GAP | 1150 | 0.750 | 0.065 | 3 | 6 | Proteobacteria(100) | Gammaproteobacteria(100) | Pseudomonadales(100) | Pseudomonadaceae(100) | Cellvibrio(100) |
| GAP | 1629 | 0.750 | 0.071 | 3 | 7 | Gemmatimonadetes(100) | Gemmatimonadetes(100) | Gemmatimonadales(100) | Gemmatimonadaceae(100) | Gemmatimonas(100) |
| GAP | 2561 | 0.750 | 0.072 | 3 | 5 | unclassified(100) | unclassified(100) | unclassified(100) | unclassified(100) | unclassified(100) |
|  |  |  |  |  |  |  |  |  |  |  |
| PAP | 8836 | 1.000 | 0.006 | 4 | 5 | unclassified(100) | unclassified(100) | unclassified(100) | unclassified(100) | unclassified(100) |
| PAP | 8180 | 1.000 | 0.007 | 4 | 7 | unclassified(100) | unclassified(100) | unclassified(100) | unclassified(100) | unclassified(100) |
| PAP | 8186 | 1.000 | 0.008 | 4 | 20 | unclassified(100) | unclassified(100) | unclassified(100) | unclassified(100) | unclassified(100) |
| PAP | 7766 | 1.000 | 0.01 | 4 | 10 | Proteobacteria(100) | Alphaproteobacteria(100) | Rhodospirillales(100) | Rhodospirillaceae(100) | Skermanella(100) |
| PAP | 1156 | 0.853 | 0.04 | 8 | 34 | Actinobacteria(100) | Actinobacteria(100) | Actinomycetales(100) | Micromonosporaceae(100) | unclassified(100) |
| PAP | 1775 | 0.833 | 0.014 | 6 | 12 | unclassified(100) | unclassified(100) | unclassified(100) | unclassified(100) | unclassified(100) |
| PAP | 554 | 0.829 | 0.046 | 7 | 35 | unclassified(89) | unclassified(89) | unclassified(89) | unclassified(89) | unclassified(89) |
| PAP | 4560 | 0.818 | 0.031 | 5 | 11 | Actinobacteria(100) | Actinobacteria(100) | unclassified(100) | unclassified(100) | unclassified(100) |
| PAP | 4620 | 0.800 | 0.03 | 5 | 5 | Actinobacteria(100) | Actinobacteria(100) | Actinomycetales(100) | Propionibacteriaceae(100) | Microlunatus(80) |
| PAP | 3490 | 0.800 | 0.039 | 7 | 20 | Actinobacteria(100) | Actinobacteria(100) | Actinomycetales(100) | unclassified(100) | unclassified(100) |
| PAP | 1980 | 0.789 | 0.022 | 7 | 19 | Acidobacteria(95) | Acidobacteria_Gp7(95) | unclassified(95) | unclassified(95) | unclassified(95) |
| PAP | 1498 | 0.776 | 0.022 | 9 | 76 | Proteobacteria(100) | Alphaproteobacteria(100) | Rhodobacterales(100) | Rhodobacteraceae(100) | Rubellimicrobium(100) |
| PAP | 5226 | 0.776 | 0.068 | 8 | 49 | unclassified(82) | unclassified(82) | unclassified(82) | unclassified(82) | unclassified(82) |
| PAP | 4366 | 0.765 | 0.023 | 5 | 17 | Verrucomicrobia(100) | Spartobacteria(100) | unclassified(100) | unclassified(100) | unclassified(100) |
| PAP | 8165 | 0.750 | 0.038 | 3 | 3 | Acidobacteria(100) | Acidobacteria_Gp7(100) | unclassified(100) | unclassified(100) | unclassified(100) |
| PAP | 7925 | 0.750 | 0.043 | 3 | 4 | unclassified(100) | unclassified(100) | unclassified(100) | unclassified(100) | unclassified(100) |
| PAP | 8356 | 0.750 | 0.047 | 3 | 5 | unclassified | unclassified | unclassified | unclassified | unclassified |
| PAP | 7753 | 0.750 | 0.048 | 3 | 8 | Actinobacteria(100) | Actinobacteria(100) | Actinomycetales(100) | unclassified(100) | unclassified(100) |
| PAP | 9031 | 0.750 | 0.049 | 3 | 5 | Actinobacteria(100) | Actinobacteria(100) | unclassified | unclassified | unclassified |
| PAP | 9144 | 0.750 | 0.049 | 3 | 7 | Bacteroidetes(100) | Sphingobacteria(100) | Sphingobacteriales(100) | Chitinophagaceae(100) | Terrimonas(100) |
| PAP | 8631 | 0.750 | 0.052 | 3 | 6 | Proteobacteria(100) | Alphaproteobacteria(100) | Sphingomonadales(84) | unclassified | unclassified |
| PAP | 8042 | 0.750 | 0.053 | 3 | 4 | Actinobacteria(100) | Actinobacteria(100) | Solirubrobacterales(100) | unclassified | unclassified |
| PAP | 8428 | 0.750 | 0.053 | 3 | 4 | Gemmatimonadetes(100) | Gemmatimonadetes(100) | Gemmatimonadales(100) | Gemmatimonadaceae(100) | Gemmatimonas(100) |
| PAP | 8674 | 0.750 | 0.053 | 3 | 5 | Actinobacteria(100) | Actinobacteria(100) | Actinomycetales(100) | Mycobacteriaceae(100) | Mycobacterium(100) |
| PAP | 1764 | 0.750 | 0.054 | 8 | 28 | Acidobacteria(100) | Acidobacteria_Gp16(100) | unclassified(100) | unclassified(100) | unclassified(100) |
| PAP | 8102 | 0.750 | 0.054 | 3 | 3 | unclassified(100) | unclassified(100) | unclassified(100) | unclassified(100) | unclassified(100) |
| PAP | 8187 | 0.750 | 0.055 | 3 | 5 | unclassified(100) | unclassified(100) | unclassified(100) | unclassified(100) | unclassified(100) |
| PAP | 8635 | 0.750 | 0.056 | 3 | 3 | Proteobacteria(100) | Alphaproteobacteria(100) | Rhodospirillales(100) | unclassified(100) | unclassified(100) |
| PAP | 7778 | 0.750 | 0.057 | 3 | 8 | unclassified(100) | unclassified(100) | unclassified(100) | unclassified(100) | unclassified(100) |
| PAP | 8794 | 0.750 | 0.057 | 3 | 3 | unclassified(100) | unclassified(100) | unclassified(100) | unclassified(100) | unclassified(100) |
| PAP | 7904 | 0.750 | 0.058 | 3 | 4 | unclassified | unclassified | unclassified | unclassified | unclassified |
| PAP | 8483 | 0.750 | 0.059 | 3 | 5 | Acidobacteria(100) | Acidobacteria_Gp6(100) | unclassified(100) | unclassified(100) | unclassified(100) |
| PAP | 8644 | 0.750 | 0.059 | 3 | 4 | unclassified(100) | unclassified(100) | unclassified(100) | unclassified(100) | unclassified(100) |
| PAP | 9457 | 0.750 | 0.059 | 3 | 3 | Proteobacteria(100) | unclassified | unclassified | unclassified | unclassified |
| PAP | 7924 | 0.750 | 0.06 | 3 | 3 | Actinobacteria(100) | Actinobacteria(100) | Actinomycetales(100) | unclassified(100) | unclassified(100) |
| PAP | 7886 | 0.750 | 0.061 | 3 | 4 | Gemmatimonadetes(100) | Gemmatimonadetes(100) | Gemmatimonadales(100) | Gemmatimonadaceae(100) | Gemmatimonas(100) |
| PAP | 9576 | 0.750 | 0.061 | 3 | 4 | unclassified(100) | unclassified(100) | unclassified(100) | unclassified(100) | unclassified(100) |
| PAP | 9713 | 0.750 | 0.061 | 3 | 4 | Actinobacteria(100) | Actinobacteria(100) | Solirubrobacterales(100) | Patulibacteraceae(100) | Patulibacter(100) |
| PAP | 8872 | 0.750 | 0.066 | 3 | 3 | Firmicutes(100) | Bacilli(100) | Bacillales(100) | Bacillaceae(100) | Bacillus(100) |
| PAP | 8704 | 0.750 | 0.067 | 3 | 4 | unclassified(100) | unclassified(100) | unclassified(100) | unclassified(100) | unclassified(100) |
| PAP | 8182 | 0.750 | 0.082 | 3 | 6 | unclassified(100) | unclassified(100) | unclassified(100) | unclassified(100) | unclassified(100) |
|  |  |  |  |  |  |  |  |  |  |  |
| NE | 3782 | 0.875 | 0.005 | 5 | 8 | Bacteroidetes(100) | Sphingobacteria(100) | Sphingobacteriales(100) | Sphingobacteriaceae(100) | Mucilaginibacter(100) |
| NE | 2989 | 0.824 | 0.014 | 7 | 17 | unclassified(100) | unclassified(100) | unclassified(100) | unclassified(100) | unclassified(100) |
| NE | 7041 | 0.818 | 0.023 | 6 | 11 | Proteobacteria(100) | unclassified(91) | unclassified(91) | unclassified(91) | unclassified(91) |
| NE | 12025 | 0.750 | 0.039 | 3 | 10 | Acidobacteria(100) | Acidobacteria_Gp6(100) | unclassified(100) | unclassified(100) | unclassified(100) |
| NE | 12386 | 0.750 | 0.053 | 3 | 8 | unclassified | unclassified | unclassified | unclassified | unclassified |
| NE | 12412 | 0.750 | 0.053 | 3 | 6 | Actinobacteria(100) | Actinobacteria(100) | Actinomycetales(100) | unclassified(100) | unclassified(100) |
| NE | 12789 | 0.750 | 0.054 | 3 | 10 | Proteobacteria(100) | Alphaproteobacteria(100) | Sphingomonadales(100) | Sphingomonadaceae(100) | unclassified |
| NE | 11813 | 0.750 | 0.056 | 3 | 6 | Actinobacteria(100) | Actinobacteria(100) | Actinomycetales(100) | Microbacteriaceae(100) | unclassified(100) |
| NE | 12004 | 0.750 | 0.058 | 3 | 9 | Actinobacteria(100) | Actinobacteria(100) | Actinomycetales(100) | Kineosporiaceae(100) | Kineosporia(100) |
| NE | 11843 | 0.750 | 0.066 | 3 | 5 | Proteobacteria(100) | Gammaproteobacteria(100) | Xanthomonadales(100) | Xanthomonadaceae(100) | Dyella(100) |
| NE | 11922 | 0.750 | 0.067 | 3 | 9 | unclassified(100) | unclassified(100) | unclassified(100) | unclassified(100) | unclassified(100) |
